# Supplementary material for: Probiotic acoustic biosensors for noninvasive imaging of gut inflammation
Source: Nat Commun. 2025 Aug 25;16:7931. doi: 10.1038/s41467-025-62569-1 (PMC12379287; doi:10.1038/s41467-025-62569-1)
Supplement: Supplementary file 1 — Supplementary Information [file 41467_2025_62569_MOESM1_ESM.pdf]

Supplementary Information
for
Probiotic acoustic biosensors for noninvasive imaging of gut inflammation

Authors: Marjorie T. Buss[1], Lian Zhu[1], Jamie H. Kwon[2], Jeffrey J. Tabor[3,4,5], Mikhail G. Shapiro[1,6,7]

Affiliations:
[1]Division of Chemistry and Chemical Engineering, California Institute of Technology, Pasadena, CA, US
[2]Division of Biology and Biological Engineering, California Institute of Technology, Pasadena, CA, US
[3]Ph.D. Program in Systems, Synthetic, and Physical Biology, Rice University, Houston, TX, USA
[4]Department of Biosciences, Rice University, Houston, TX, USA
[5]Department of Bioengineering, Rice University, Houston, TX, USA
[6]Andrew and Peggy Cherng Department of Medical Engineering, California Institute of Technology, Pasadena, CA, US
[7]Howard Hughes Medical Institute, California Institute of Technology, Pasadena, CA, US

Supplementary Information Contents:
Supplementary Tables S1-3
Supplementary Figures S1-S22
Supplementary Note 1
Supplementary Video Captions
Supplementary References

Table S1: Sequences of optimized thiosulfate and tetrathionate sensor components.

Description	Sequence (5’ -> 3’)
Variant *thsS*(t3) with improved performance at 37°C<br><br>(point mutations highlighted)	ATGTCCCGCCTGCTGCTGTGTATCTGTGTTCTGCTGTTCTCTTCTGTGGCGT<br>GGTCTAAACCGCAGCAGTTTTATGTGGGCGTACTGGCTAACTGGGGTCATCA<br>GCAAGCCGTTGAACGTTGGACCCCGATGATGGAGTATCTGAACGAACACGT<br>GCCGGACGCGGAATTTCACGTCTACCCGGGCAACTTCAAAGCACTGAACCT<br>GGCAATGGAACTGGGCCAGATTCAGTTCATTATCACTAACCCGGGCCAATATC<br>TGTACCTGAGCAATCAGTACCCGCTGTCTTGGCTGGCGACCATGCGTTCTAA<br>GCGTCACGATGGTACCACTTCTGCGATCGGTTCCGCCATTATTGTCCGCGCG<br>GACAGCGACTACCGCACCCTGTACGACCTGAAAGGTAAAGTGGTGGCTGCG<br>TCCGACCCGCATGCTCTGGGTGGCTACCAAGCGACCGTCGGTCTGATGCAT<br>TCCCTGGGCATGGATCCGGACACCTTCTTCGGTGAAACCAAGTTTCTGGGCT<br>TTCCACTGGATCCGCTGCTGTACCAAGTTCGTGATGGCAACGTTGACGCGGC<br>CATTACCCCACTGTGCACTCTGGAGGACATGGTTGCACGCGGCGTACTGAAA<br>TCTTCCGATTTTCGTGTGCTGAACCCTAGCCGCCCGGATGGTGTAGAATGCC<br>AGTGCTCTACCACCCTGTACCCGAACTGGTCTTTCGCTGCGACTGAGTCTGT<br>ATCCACCGAACTGTCTAAAGAAATCACGCAGGCACTGCTGGAACTGCCATCC<br>GACAGCCCGGCAGCTATCAAAGCGCAACTGACCGGCTGGACCAGCCCGATC<br>TCCCAACTGGCGGTAATCAAACTGTTCCAAGAGCTGCACGTAAAAACCCCGG<br>ACTCTAGCCGTTGGGAAGCCGTTAAGAAGTGGCTGGAAGAAAACCGTCACT<br>GGGGTATCCTGTCTGTTCTGGTGTTCATCATTGCAACGCTGTATCACCTGTGG<br>ATTGAATACCGCTTCCACCAAAAAAGCTCCTCTCTGATCGAATCTGAACGTCA<br>GCTGAAACAGCATGCTGTTGCCCTGGAACGTCTGCAATCTGCTAGCATCGTT<br>GGTGAAATTGGTGCGGGTCTGGCCCACGAGATTAATCAGCCGATCGCTGCAA<br>TTACCTCTTATTCTGAAGGTGGCATCATGCGCCTGCAAGGTAAAGAACAGGC<br>GGATACGGATAGCTGCATCGAACTGCTGGAAAAAATCCACAAACAGAGCACT<br>CGCGCAGGCGAAGTGGTGCACCGCATCCGTGGTCTGCTGAAACGTCGTGAA<br>GCGGTGATGGTAGATGTTAACATCCTGACCCTGGTGGAAGAATCCATCAGCC<br>TGCTGCGTCTGGAGCTGGCACGTCGCGAAATCCAGATCAACACTCAGATCAA<br>AGGTGAACCGTTCTTCATTACTGCCGACCGCGTTGGCCTGCTGCAAGTTCTG<br>ATTAACCTGATCAAAAACTCCCTGGACGCGATCGCTGAATCTGATAATGCCCG<br>TTCTGGTAAAATCAACATCGAACTGGACTTTAAAGAGTACCAGGTAAACGTCT<br>CCATCATCGATAACGGTCCGGGCCTGGCGATGGATTCTGACACTCTGATGGC<br>TACGTTTTACACTACCAAAATGGATGGCCTGGGCCTGGGTCTGGCAGTCTGC<br>CGCGAAGTTATCAGCAACCACGACGGCCACATCCTGCTGTCCAACCGTGAC<br>GACGGCGTTCTGGGCTGTGTGGCAACCCTGAATCTGAAAAAACGCGGTTCT<br>GAAGTGCCGATCGAAGTCTAA
Constitutive promoter for *ttrR* in variant m13<br><br>(point mutations highlighted)	TTGATAGCTAGCTCAGTCCTAGGTATTGTGCTAGC

Table S2: Parameters for fitting sensor characterization data from Fig. 3 and Fig. S6 to the Hill equation:

$$Y = A + \frac{B}{1 + \left(\frac{K}{x}\right)^n}$$

Here, Y is the sensor output (ultrasound signal or fluorescence) at the ligand (thiosulfate or tetrathionate) concentration x in μM. A, B, K, and n are constants where A is the minimum output with no ligand, B is the maximum output with saturating ligand concentration, K is ligand concentration that elicits a half-maximal response, and n is the Hill coefficient. Parameters are reported as the fitted value ± the standard error.

Output	BURST SBR		xAM SBR	
Strain	thsS(t3)R-bARG$_{Ser}$	thsS(t3)R-Bxb1_P7-bARG$_{Ser}$	thsS(t3)R-bARG$_{Ser}$	thsS(t3)R-Bxb1_P7-bARG$_{Ser}$
A	14.96 ± 0.9762	4.043 ± 0.3336	1.017 ± 0.01991	1.081 ± 0.03498
B	152.1 ± 10.20	321.4 ± 16.83	5.416 ± 0.1221	12.88 ± 0.3666
K	76.42 ± 6.386	48.23 ± 4.128	72.53 ± 2.080	54.93 ± 2.247
n	2.912 ± 0.4397	2.126 ± 0.1304	3.002 ± 0.1860	2.654 ± 0.1585
R^2	0.8769	0.9336	0.9834	0.9760

Output	Mean GFP Fluorescence	
Strain	thsS(t3)R-GFP	thsS(t3)R-Bxb1_P7-GFP_mCherry
A	188.8 ± 5.021	135.3 ± 11.75
B	3580 ± 104.9	39473 ± 2254
K	105.0 ± 2.756	57.20 ± 3.942
n	4.634 ± 0.2870	2.790 ± 0.1454
R^2	0.9701	0.9180

Output	BURST SBR		xAM SBR	
Strain	ttrSR(m13)-bARG$_{Ser}$	ttrSR(m13)-Bxb1_P7-bARG$_{Ser}$	ttrSR(m13)-bARG$_{Ser}$	ttrSR(m13)-Bxb1_P7-bARG$_{Ser}$
A	8.280 ± 0.5146	34.57 ± 1.830	1.034 ± 0.01942	2.124 ± 0.04621
B	145.2 ± 10.37	203.7 ± 17.02	4.782 ± 0.1206	12.28 ± 0.4240
K	32.77 ± 5.688	44.56 ± 7.114	25.58 ± 1.361	41.98 ± 2.996
n	1.205 ± 0.1125	1.646 ± 0.2303	1.945 ± 0.1185	1.490 ± 0.08407
R^2	0.9367	0.8859	0.9842	0.9801

Output	Mean GFP Fluorescence	
Strain	ttrSR(m13)-GFP	ttrSR(m13)-Bxb1_P7-GFP_mCherry
A	57.80 ± 1.068	143.6 ± 5.553
B	916.1 ± 18.95	31452 ± 1046
K	34.53 ± 1.200	27.84 ± 1.271
n	2.328 ± 0.08339	2.981 ± 0.1149
R^2	0.9896	0.9668

Table S3: Sequence verification of genomic modifications to EcN.

**Description**	**Sequence (5' -> 3')**
Fwd primer1	CCAGCCAGATGGCCTGG
Rev primer1	GACGCGACGACGTGGC
Colony PCR spontaneous streptomycin-resistant EcN with Fwd primer1 and Rev primer1<br><br>*rpsL* underlined<br><br>Point mutation in *rpsL* highlighted	GGAGCTATTTAATGGCAACAGTTAACCAGCTGGTACGCAAACCACGTGCT<br>CGCAAAGTTGCGAAAAGCAACGTGCCTGCGCTGGAAGCATGCCCGCAA<br>AAACGTGGCGTATGTACTCGTGTATATACTACCACTCCTACAAAACCGAAC<br>TCCGCGCTGCGTAAAGTATGCCGTGTTCGTCTGACTAACGGTTTCGAAGT<br>GACTTCCTACATCGGTGGTGAAGGTCACAACCTGCAGGAGCACTCCGTG<br>ATCCTGATCCGTGGTGGTCGTGTTAAAGACCTCCCGGGTGTTCGTTACC<br>ACACCGTACGTGGTGCGCTTGACTGCTCCGGCGTTAAAGACCGTAAGCA<br>GGCTCGTTCCAAGTATGGCGTGAAGCGTCCTAAGGCTTAATGGTTCTCC<br>GTTAAGTAAGGCCAAACGTTTTAACTTAAATGTCAAACTAAACTCGTAGAG<br>TTTTGGACAATCCTGAATTAACAACGGAGTATTTCCATGCCACGTCGTCG<br>CGTCA
Fwd primer2	GGAATCAATGCCTGAGTG
Rev primer2	GGCATCAACAGCACGTTC
Colony PCR EcN att$_{HK}$::bla$_{TEM1\text{-}B}$ with Fwd primer 2 and Rev primer 2<br><br>**J23119 promoter bolded**<br><br>bla$_{TEM1\text{-}B}$ underlined	GTTTACCGTGGCTCCATTGCACCAGATATCGTCCTGGTTGATAGCCCATC<br>TGAAACCAGGGCACACCAACGCGGCTTTTCACGTGGGGAGCATCAATAG<br>CATTCACCAGTTCGATCACCGGCAGACTTGCTACCTGCTTTTGCAGATCG<br>GGAAATGACGTCGTGCTACTACCGAGCAAAATGGCCTCTGCGCCCCACT<br>GTTTACACTGGTCGATTTGTGCTTGCTGCGTAGCCAACTGGCTGTAGCC<br>GCCTGCCTCCAGCACTTTTAAATCCACACCGTAGCGGCGAGCAGCCTCC<br>TGCATACCATAGTTCAACGATAACCAGTATGAATCTTTCAGGCTGGGATAA<br>AGCGCGCACAGTTTCCATGCGCGTTTGGCTTTAAGCGGCGTAGAGGCTT<br>GCACCGTGTAATGCTGCGCATCATGCCAGCGCAACAGGTTATCAGCCGA<br>AAATGCCGATAACATGAAAAGGGAAAGAAGTAAAAATAGCAGTACGCGCA<br>TGATAGCCTCATCAATAATAAGGCTTTATGCTAGATGCATTCTGCTTTGCGA<br>CTCAACCTTTTTCACCTAAAGGATGACAAAATAACATTAATCACTTAAAAAT<br>CATCGCATTACACTAATCTGTGGTTAAATGATAGACTACATAATGCGACAAA<br>ACGCAACATATCCAGTCACTATGAATCAACTACTTAGATAGTATTAGTGACC<br>TGAGACAGAGCATTAGCTAGCTTCTTCGTCTGTTTCTACTGGTATTGGCA<br>CAAACCTGATTCCAATTTGAGCAAGGCTATGTGCCATCTCGATACTCGTTC<br>TTAACTCAACAGAAGATGCTTTGTGCATACAGCCCCTCGTTTATTATTTATC<br>TCCTCAGCCAGCCGCTGTGCTTTCAGTGGATTTCGGATAACAGAAAGGC<br>CGGGAAATACCCAGCCTCGCTTTGTAACGGAGTAGAGACGAAAGTGATT<br>GCGCCTACCCGGATATTATCGTGAGGATGCGTCATCGCCATTAATTCACT<br>GATCAGTGATAAGCTGTCAAACATGAGAATTGATCCGGCTGCCTCGCGC<br>GTTTCGGTGATGACGGTGAAAACCTCTGACACATGCAGCTCCCGGAGAC<br>GGTCACAGCTTGTCTGTAAGCGGATGCCGGGAGCAGACAAGCCCGTCA<br>GGGCGCGTCAGCGGGTGTTGGCGGGTGTCGGGGCGCAGCCATGACCC<br>AGTCACGTAGCGATAGCGGAGTGTATGCTGCACATGACATTAACCTATAAA<br>AATAGGCGTATCACGAGGCCCTTTCGTCTTCAAGAATTAATTCCCAATTCC<br>CCAGGCATCAAATAAAACGAAAGGCTCAGTCGAAAGACTGGGCCTTTCG<br>TTTTATCTGTTGTTTGTCGGTGAACGCTCTCCTGAGTAGGACAAATCCGC<br>CGGGAGCGGATTTGAACGTTGCGAAGCAACGGCCCGGAGGGTGGCGG<br>GCAGGACGCCCGCCATAAACTGCCAGGAATTAATTCCCCAGGCATCAAAT<br>AAAACGAAAGGCTCAGTCGAAAGACTGGGCCTTTCGTTTTATCTGTTGTT<br>TGTCGGTGAACGCTCTCCTGAGTAGGACAAATCCGCCGGGAGCGGATTT<br>GAACGTTGCGAAGCAACGGCCCGGAGGGTGGCGGGCAGGACGCCCGC<br>CATAAACTGCCAGGAATTAATTCCCCAGGCATCAAATAAAACGAAAGGCT<br>CAGTCGAAAGACTGGGCCTTTCGTTTTATCTGTTGTTTGTCGGTGAACGC<br>TCTCCTGAGTAGGACAAATCCGCCGGGAGCGGATTTGAACGTTGCGAAG<br>CAACGGCCCGGAGGGTGGCGGGCAGGACGCCCGCCATAAACTGCCAG<br>GAATTGGGGATCGGCGTTTCTACAAACTCT**TTGACAGCTAGCTCAGTCCT**

	**AGGTATAATGCTAGC**AAAGAGGAGAAATAATAGATGAGTATTCAACATTTT<br>CGTGTCGCCCTTATTCCCTTTTTTGCGGCATTTTGCCTTCCTGTTTTTGCT<br>CACCCAGAAACGCTGGTGAAAGTAAAAGATGCTGAAGATCAGTTGGGTG<br>CACGAGTGGGTTACATCGAACTGGATCTCAACAGCGGTAAGATCCTTGA<br>GAGTTTTCGCCCCGAAGAACGTTTTCCAATGATGAGCACTTTTAAAGTTC<br>TGCTATGTGGTGCGGTATTATCCCGTGTTGACGCCGGGCAAGAGCAACT<br>CGGTCGCCGCATACACTATTCTCAGAATGACTTGGTTGAGTACTCACCAG<br>TCACAGAAAAGCATCTTACGGATGGCATGACAGTAAGAGAATTATGCAGT<br>GCTGCCATAACCATGAGTGATAACACTGCTGCCAACTTACTTCTGACAAC<br>GATCGGAGGACCGAAGGAGCTAACCGCTTTTTTGCACAACATGGGGGAT<br>CATGTAACTCGCCTTGATCGTTGGGAACCGGAGCTGAATGAAGCCATACC<br>AAACGACGAGCGTGACACCACGATGCCTGCAGCAATGGCAACAACGTTG<br>CGCAAACTATTAACTGGCGAACTACTTACTCTAGCTTCCCGGCAACAATTA<br>ATAGACTGGATGGAGGCGGATAAAGTTGCAGGACCACTTCTGCGCTCGG<br>CCCTTCCGGCTGGCTGGTTTATTGCTGATAAATCTGGAGCCGGTGAGCG<br>TGGGTCTCGCGGTATCATTGCAGCACTGGGGCCAGATGGTAAGCCCTCC<br>CGTATCGTAGTTATCTACACGACGGGGAGTCAGGCAACTATGGATGAACG<br>AAATAGACAGATCGCTGAGATAGGTGCCTCACTGATTAAGCATTGGTAAC<br>AAAAAAAAACCCCGCCCCTGACAGGGCGGGGTTTTTTTTCAGGCATTTG<br>AGAAGCACACGCATGCCTCGAGATGCATGGCGCCTAACCTAAACTGACA<br>GGCATCAAATTAAGCAGAAGGCCATCCTGACGGATGGCCTTTTTGCGTTT<br>CGAACAATTGAAAAAACCTCGCGCCTTACCTGTTGAGTAATAGTCAAAAG<br>CCTCCGGTCGGAGGCTTTTGACTTTCTGCTTACTGAATTTCGGTGGTGC<br>CGTTAATTAACCGGTGGGCCCTCATGATAATAATGGTTTCTTAGACGTCCG<br>AAGTTCCTATTCTCTAGAAAGTATAGGAACTTCCATATGCCATGGGACAAA<br>ATTGAAATCGACAAATGATTTTATTTTGACTAATAATGACCTACTTACATTAA<br>TTTACTGATAATTAAAGAGATTTTAAATATACAACTTATTCACCTAAAGTGCA<br>CCGACCGTGAATTTAACCCTGACCCGAAGACTCTGGATGGGCTTTGCCC<br>TGATGGCGCTGTTAACCCTGACCAGTACCCTGGTGGGATGGTACAACCT<br>GCGCTTTATCAGCCAGGTGGAAAAA

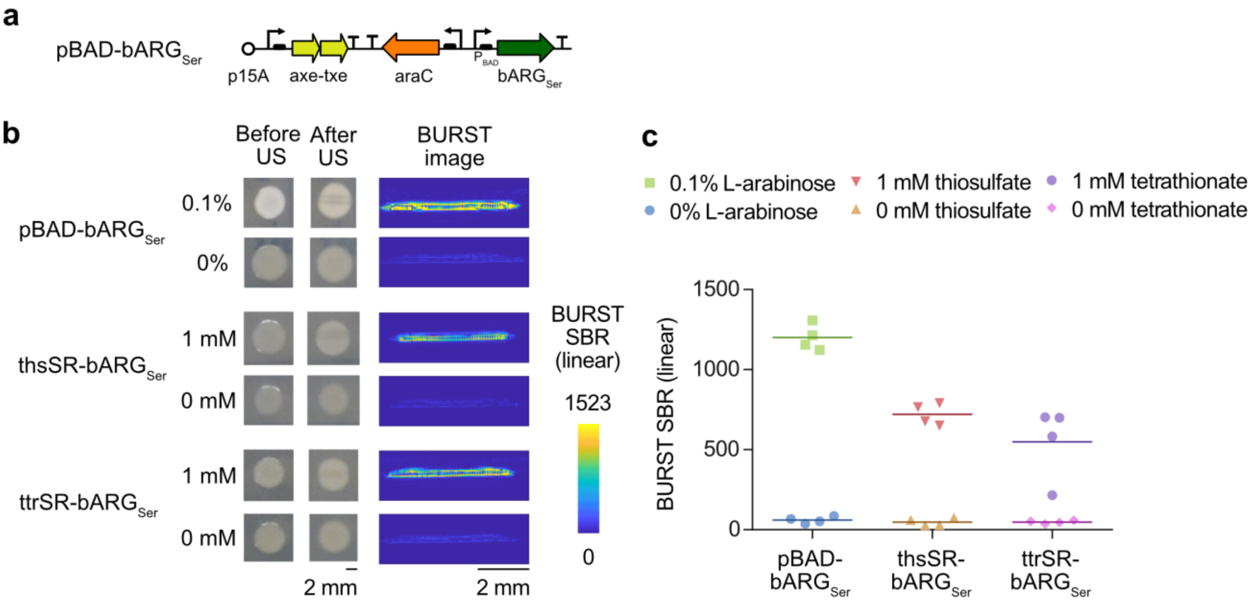

Figure S1: Arabinose-inducible versus initial thiosulfate and tetrathionate sensor constructs. (**a**) Plasmid diagram of the arabinose-inducible $bARG_{Ser}$ construct pBAD-$bARG_{Ser}$. (**b**) Images of patches of EcN strains containing the arabinose-inducible construct pBAD-$bARG_{Ser}$, the initial thiosulfate sensor construct thsSR-$bARG_{Ser}$, or the initial tetrathionate sensor construct ttrSR-$bARG_{Ser}$ (see Fig. 2a for plasmid maps) on M9 plates without inducer or with 0.1% L-arabinose, 1 mM thiosulfate, or 1 mM tetrathionate, respectively. Photographs show the opacity of representative patches before ultrasound (US) imaging (left) or after being covered with agar and imaged using BURST ultrasound which reduces opacity along the imaging plane due to GV collapse (middle). Representative BURST images (right) show the ARG-specific ultrasound signal in cross-sections of the EcN patches. (**c**) Quantification of BURST images in terms of signal-to-background ratio (SBR). Points represent biological replicates (N=4) and lines represent the mean.

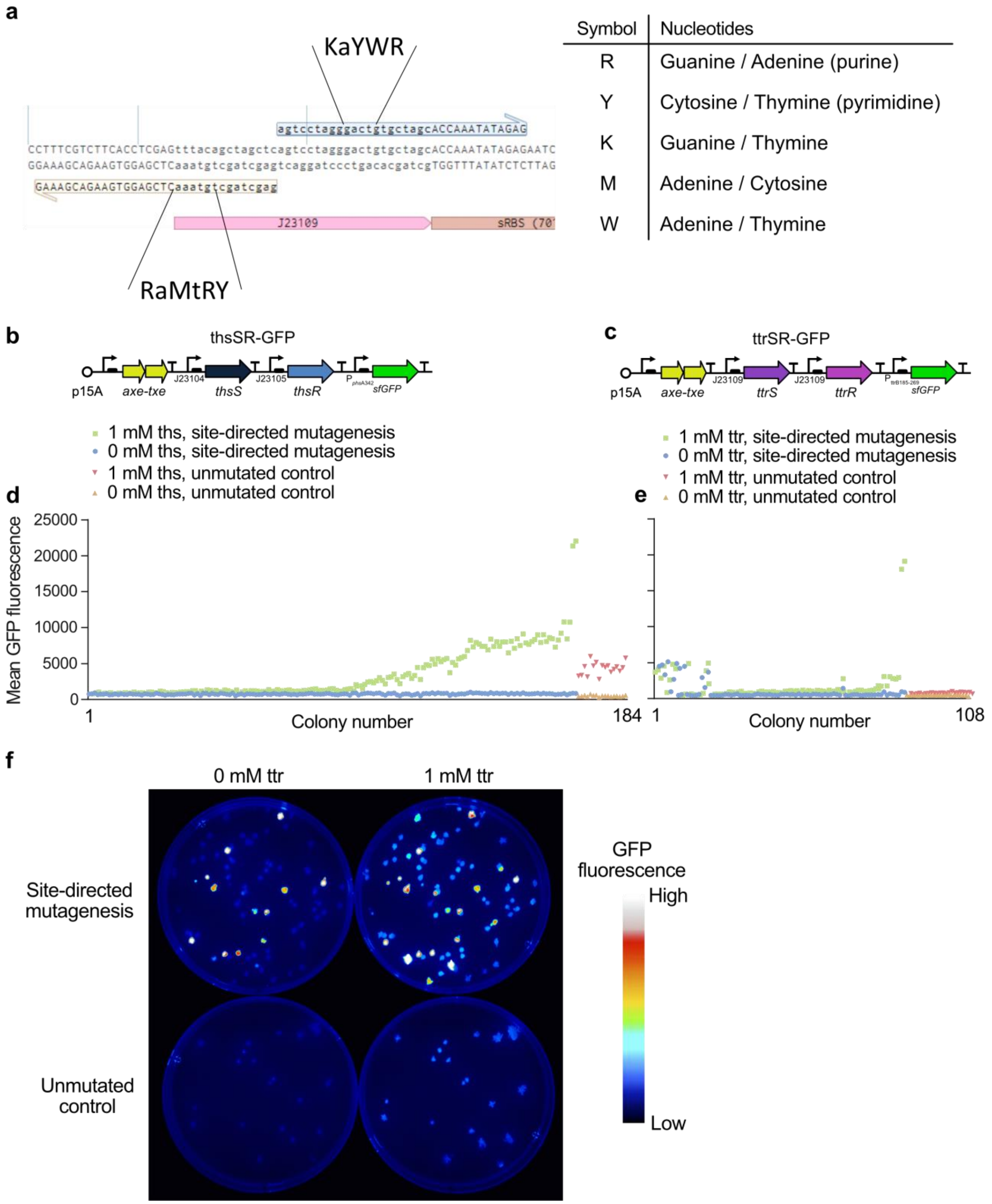

Figure S2: Site-directed mutagenesis and screening of GFP versions of thiosulfate and tetrathionate sensors. (**a**) Diagram of semi-random primers used for PCR for site-directed mutagenesis of the response regulator promoter, here J23109. The semi-random bases were chosen to broadly sample the Anderson promoter library[1]. (**b-c**) Plasmid diagrams of thiosulfate (thsSR-GFP) and tetrathionate (ttrSR-GFP) sensors with GFP as the output. (**d-e**) Mean GFP fluorescence of NEB Stable *E. coli* colonies from replica plating transformants on plates with 0 or 1 mM thiosulfate (ths) or tetrathionate (ttr) at 30°C after performing site-

directed mutagenesis on the response regular promoter (site-directed mutagenesis) or leaving the plasmid unmutated (unmutated control). (d-e) Share the same y-axis values. Points represent the mean fluorescence of single colonies. (**f**) Representative GFP fluorescence images of replica plates of mutant library (top) or unmutated control (bottom) of ttrSR-GFP with 0 or 1 mM ttr.

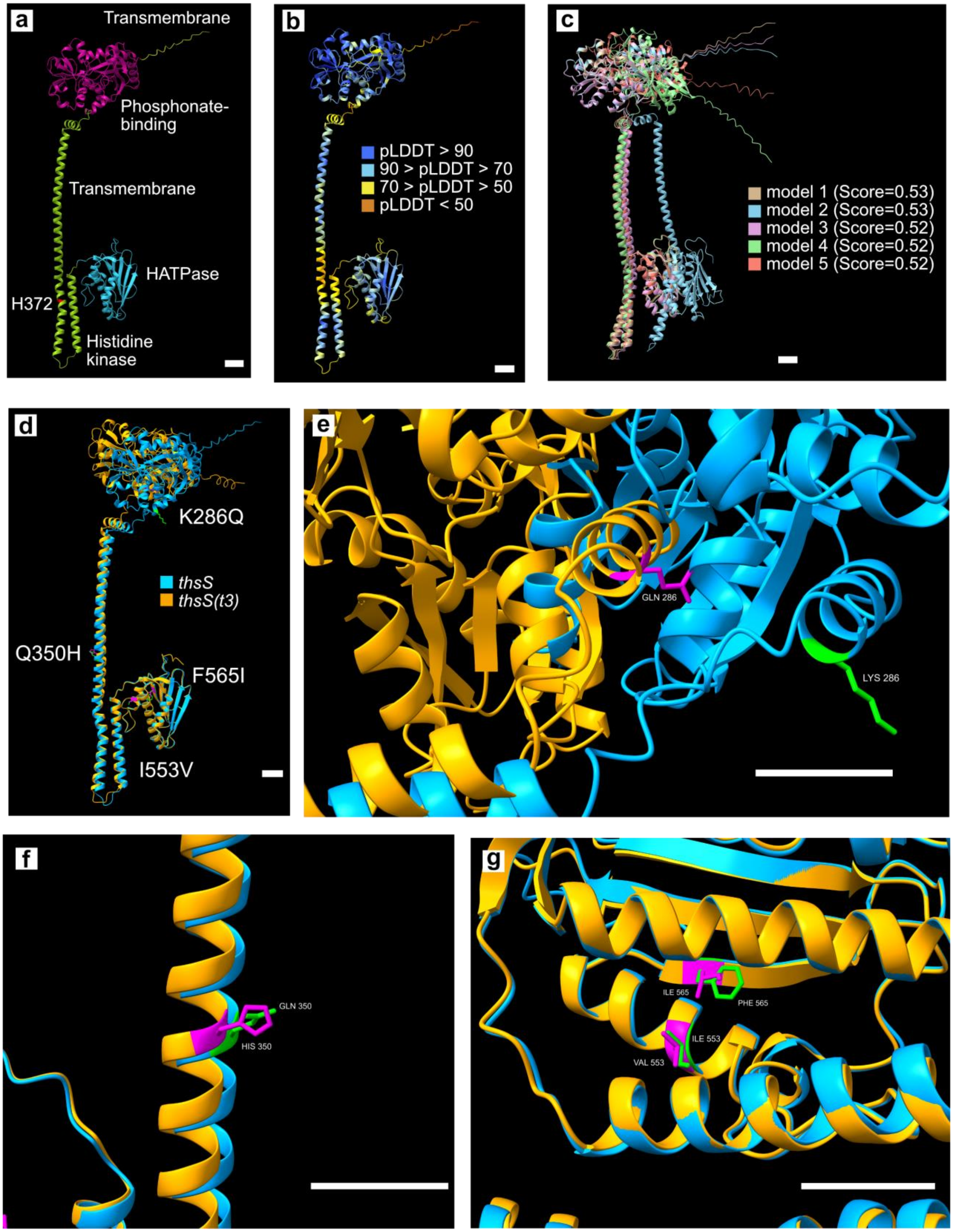

Figure S3: Structural predictions of the thiosulfate membrane sensor kinase protein *thsS* from *Shewanella halifaxensis* using AlphaFold 3[Ref. 2]. (**a**) Highest-ranked model of *thsS* colored by the

predicted domains[3]. The histidine predicted to be involved in phospho-transfer is indicated in red (H372). (**b**) Highest-ranked model of *thsS* colored by the confidence score in terms of the pLDDT (predicted local distance difference test). (**c**) Alignment of the top-five highest ranked models of *thsS* and their corresponding overall ranking score. (**d**) Alignment of the highest-ranked models for *thsS* and the *thsS(t3)* variant which was identified in the screen depicted in Fig. 2d. The four amino acids that are different between the *thsS* and *thsS(t3)* are indicated in green and magenta, respectively. (**e-g**) Close-up views of the K286Q (e), Q350H (f), and I553V & F565I (g) point mutations from the structures depicted in (d). All scale bars are 10 Å.

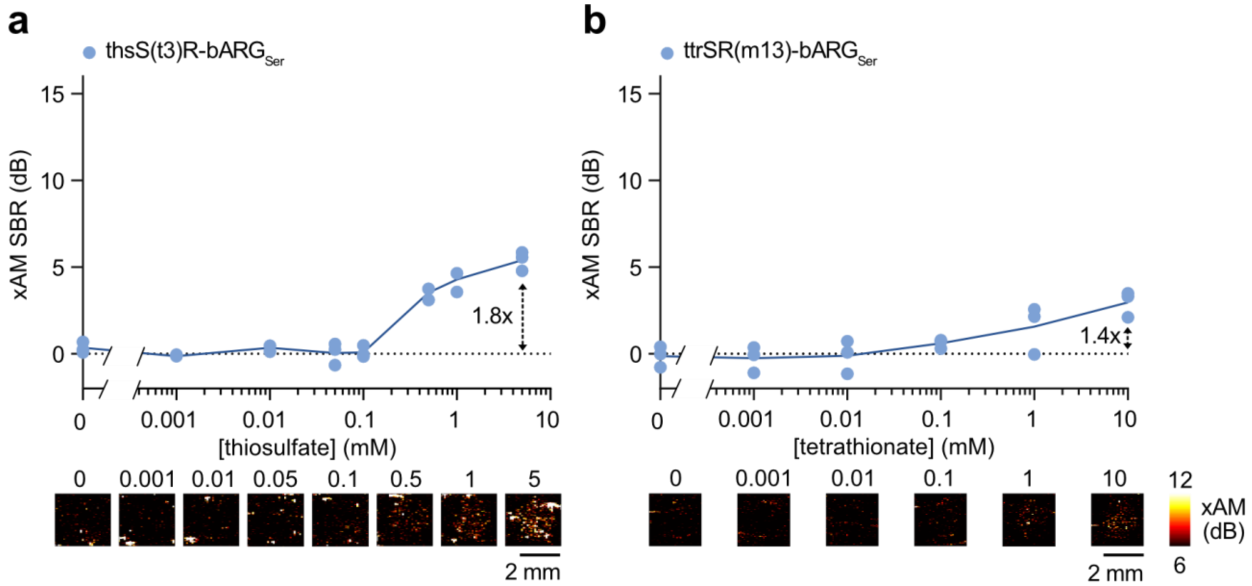

Figure S4: xAM ultrasound imaging of optimized thiosulfate and tetrathionate sensors. (**a-b**) Representative xAM ultrasound images (bottom) and quantification of the signal-to-background ratio (SBR) (top) of the best variants for thsSR-bARG$_{Ser}$ (a) and ttrSR-bARG$_{Ser}$ (b) at varying thiosulfate/tetrathionate concentrations in EcN at 37°C in liquid culture. Cells were cast in agarose phantoms at 10^9 cells/mL for ultrasound imaging. Points represent biological replicates (N=3, which were each averaged over 2 technical replicates) and solid lines connect the means.

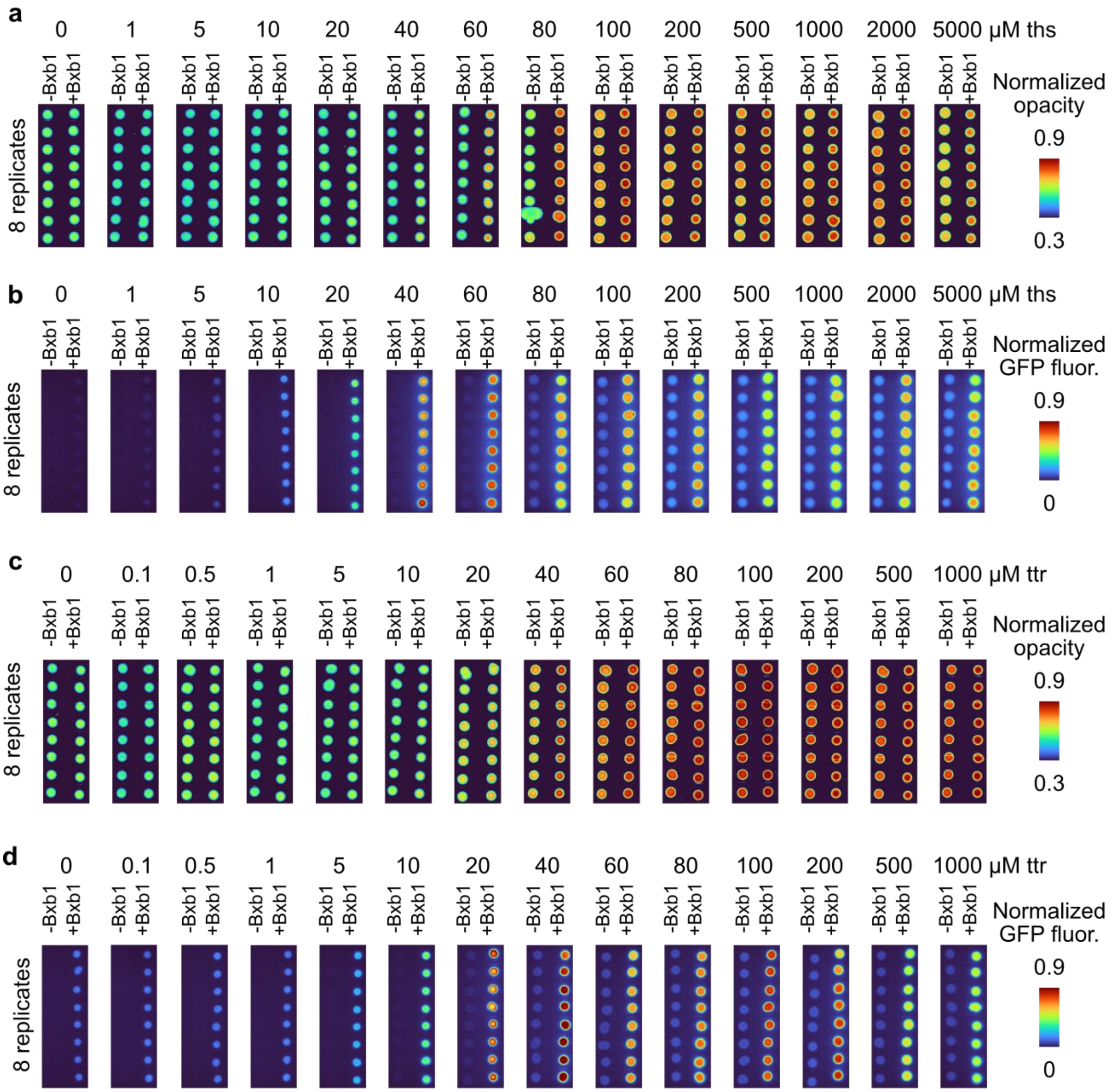

Figure S5: Images of patches of EcN sensor strains with and without the Bxb1 switch on plates with varying thiosulfate and tetrathionate concentrations. (**a**) Normalized transmitted white light images showing the opacity of thsS(t3)R-bARG$_{Ser}$ (-Bxb1) and thsS(t3)R-Bxb1_P7-bARG$_{Ser}$ (+Bxb1) EcN patches grown on plates at varying thiosulfate concentrations. (**b**) Normalized green fluorescence images of thsS(t3)R-GFP_mCherry (-Bxb1) and thsS(t3)R-Bxb1_P7-GFP_mCherry (+Bxb1) EcN patches grown on plates at varying thiosulfate concentrations. (**c**) Normalized transmitted white light images showing the opacity of ttrSR(m13)-bARG$_{Ser}$ (-Bxb1) and ttrSR(m13)-Bxb1_P7-bARG$_{Ser}$ (+Bxb1) EcN patches grown on plates at varying tetrathionate concentrations. (**d**) Normalized green fluorescence images of ttrSR(m13)-GFP_mCherry (-Bxb1) and ttrSR(m13)-Bxb1_P7-GFP_mCherry (+Bxb1) EcN patches grown on plates at varying tetrathionate concentrations. All patches were grown on M9 plates at 37°C. Patches were suspended in PBS for the ultrasound imaging and flow cytometry depicted in Fig. 3 and S6.

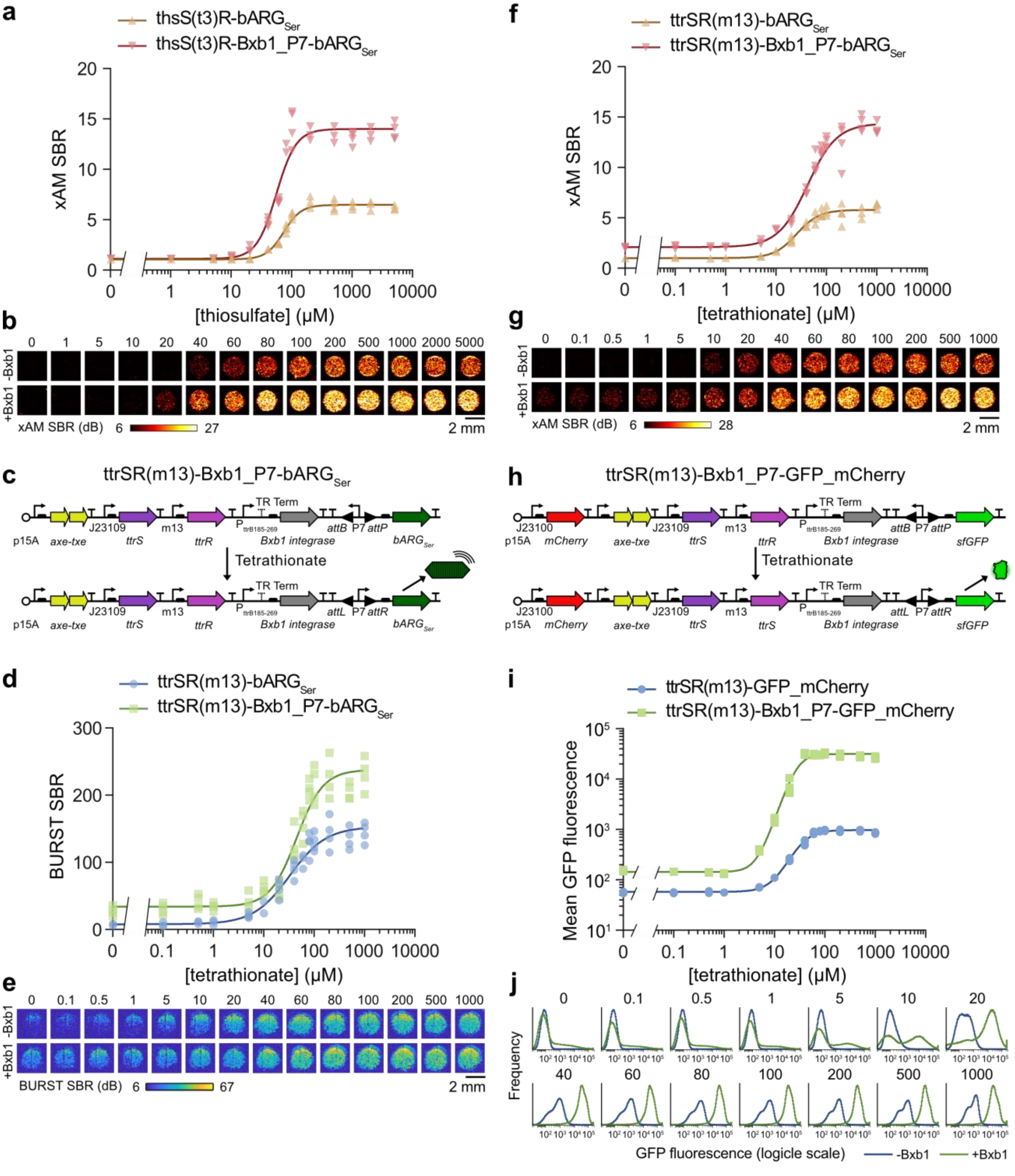

Figure S6: Additional in vitro characterization data for integrase-based switch sensors. (**a-b**) xAM signal-to-background ratio (SBR) (a) and representative images (b) of the optimized thiosulfate sensor with and without the Bxb1 integrase-based switch at varying thiosulfate concentrations. See Fig. 3b-c for the corresponding BURST data. (**c**) Plasmid diagram of the optimized tetrathionate sensor ttrSR(m13)-bARG$_{Ser}$ with an integrase-based switch to create ttrSR(m13)-Bxb1_P7-bARG$_{Ser}$. (**d-e**) BURST signal-to-background ratio (SBR) (d) and representative images (e) of the optimized tetrathionate sensor with and without the Bxb1 integrase-based switch at varying tetrathionate concentrations. (**f-g**) xAM signal-to-background ratio (SBR) (f) and representative images (g) of the optimized tetrathionate sensor with and

without the Bxb1 integrase-based switch at varying tetrathionate concentrations. (**h**) Plasmid diagram of the optimized tetrathionate sensor ttrSR(m13)-GFP_mCherry with an integrase-based switch to create ttrSR(m13)-Bxb1_P7-GFP_mCherry. (**i-j**) Mean GFP fluorescence measured via flow cytometry (i) and representative histograms (j) of the optimized tetrathionate sensor with and without the Bxb1 integrase-based switch at varying tetrathionate concentrations. In (a), (d), (f), and (i), points represent biological replicates (N=4) and curves represent fits to the Hill equation (see Table S2 for fitted parameters). All strains were grown on plates with varying concentrations of thiosulfate and tetrathionate at 37°C (see Fig. S5 for images of the plates) and suspended in PBS for ultrasound imaging and flow cytometry; for ultrasound imaging, cells were cast in agarose phantoms at a concentration of 5×10^8 cells/mL.

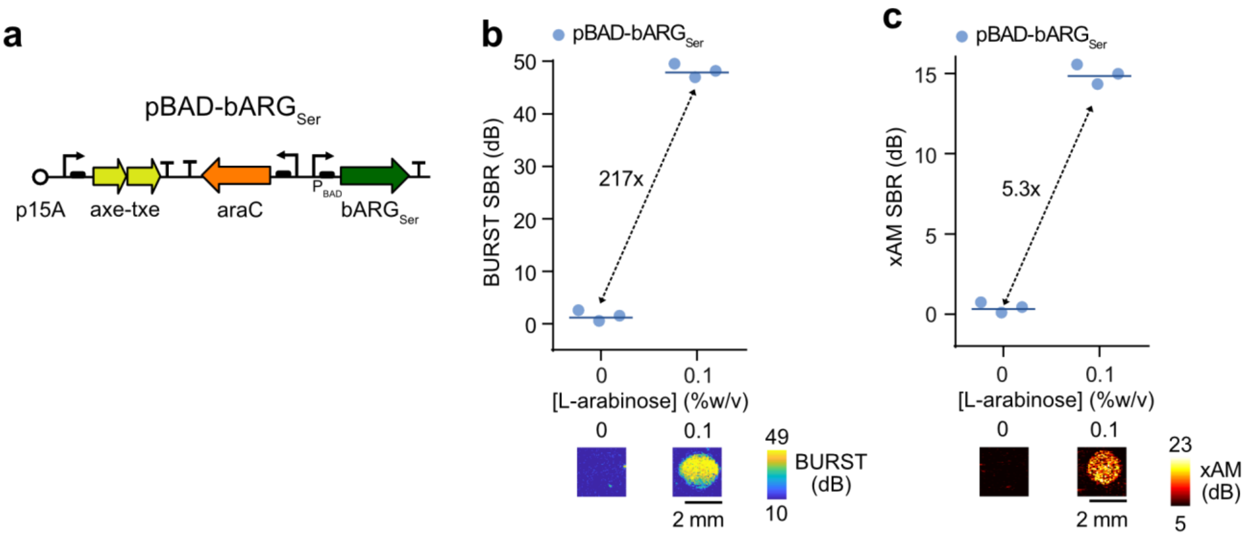

Figure S7: In vitro characterizations of arabinose-inducible bARG$_{Ser}$ in EcN. (**a**) Plasmid diagram of the arabinose-inducible bARG$_{Ser}$ construct pBAD-bARG$_{Ser}$. (**b-c**) Representative BURST (b) and xAM (c) ultrasound images (bottom) and quantification of the signal-to-background ratio (SBR) (top) of pBAD-bARG$_{Ser}$ at 0 and 0.1% L-arabinose in *E. coli* Nissle (EcN) at 37°C in liquid culture. Cells were cast in agarose phantoms at 10^9 cells/mL for ultrasound imaging. Points represent biological replicates (N=3, which were each averaged over 2 technical replicates) and solid lines represent the mean.

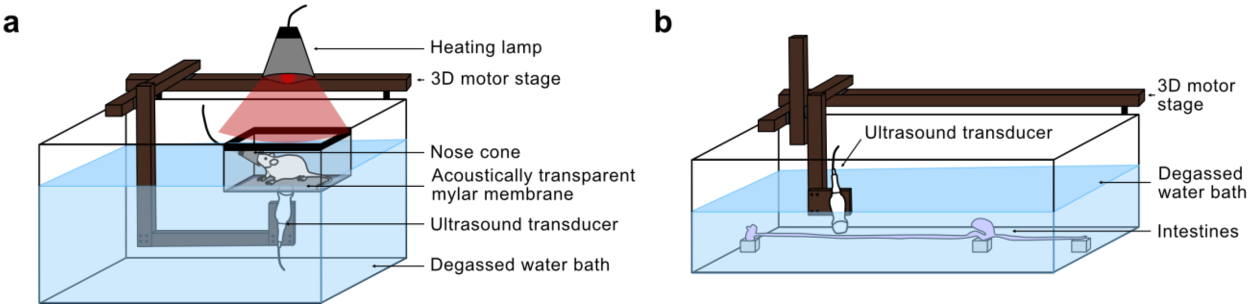

Figure S8: Custom-built in vivo and ex vivo ultrasound scanning systems. (**a-b**) Diagrams of the ultrasound imaging setups for fast and easy scanning of the entire abdominal area of live mice (a) and of the entire mouse GI tract ex vivo (b). In both setups, the 3-D translatable stage was purchased from Velmex (Motorized BiSlide Systems) and the ultrasound transducer was attached to the stage with a custom 3D-printed holder. In (a), a probe cover (Protek, part number 1-519-2450) was used to protect the ultrasound transducer so that it could be submerged underwater, and the acoustically transparent mylar film (2.5 μm thickness, Chemplex, catalogue number 100) under the mouse was secured to a plastic frame using waterproof tape (Gorilla Glue, Inc). In (b), linearized intestines were pinned using needles onto platforms (depicted as gray cubes) which consisted of acoustic absorbers (Precision acoustics). Magnets were embedded into the platforms on their bottoms so that the platforms would stay submerged and could be moved to stretch out the intestines (whose length varied between mice) using another external magnet on the bottom of the water bath. Please see methods for more details.

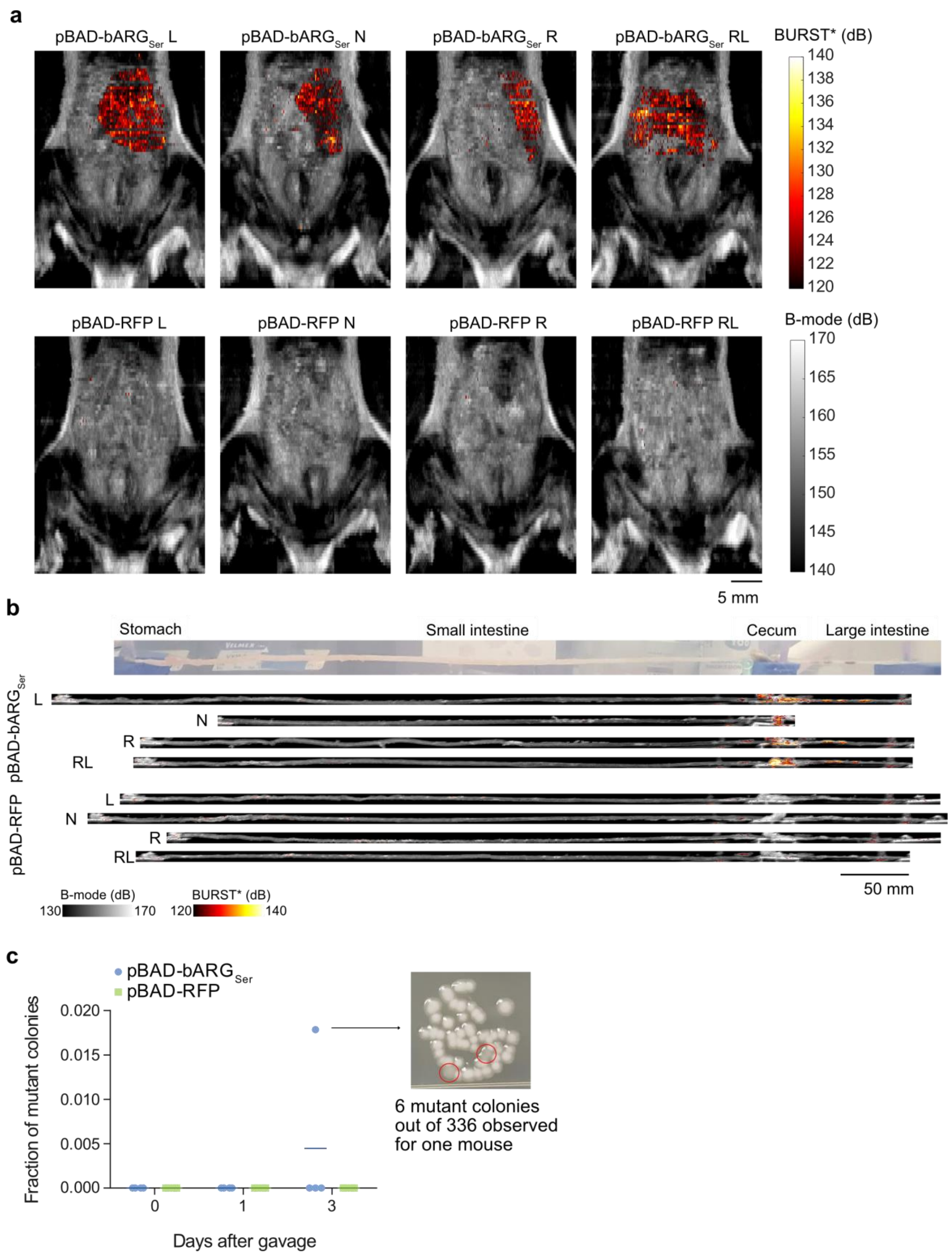

Figure S9: All replicates and additional data for imaging arabinose-inducible bARG$_{Ser}$ expression in EcN colonizing the GI tract. (**a**) Ultrasound images overlaying the integrated BURST* signal over the

depth onto the integrated B-mode signal over the depth for all mice one day after administration of the L-arabinose-sensing EcN using the setup depicted in Fig. S8a. (**b**) Ex vivo ultrasound images of intestines from all mice 3 days after administration of the L-arabinose-sensing EcN using the setup depicted in Fig. S8b. The integrated BURST* signal over the width was overlaid onto the integrated B-mode signal over the width. (**c**) Fraction of non-opaque or non-RFP-fluorescent mutant colonies detected by plating the gavage mixtures (day 0) or the feces on days 1 and 3 after gavage of the L-arabinose-sensing EcN onto plates with L-arabinose. Six non-opaque colonies (out of 336 total colonies) were observed in one mouse colonized by pBAD-bARG$_{Ser}$ EcN on day 3. Three of these colonies (red circles) are depicted in the image on the right. Points represent biological replicates (N=4) and lines represent the mean.

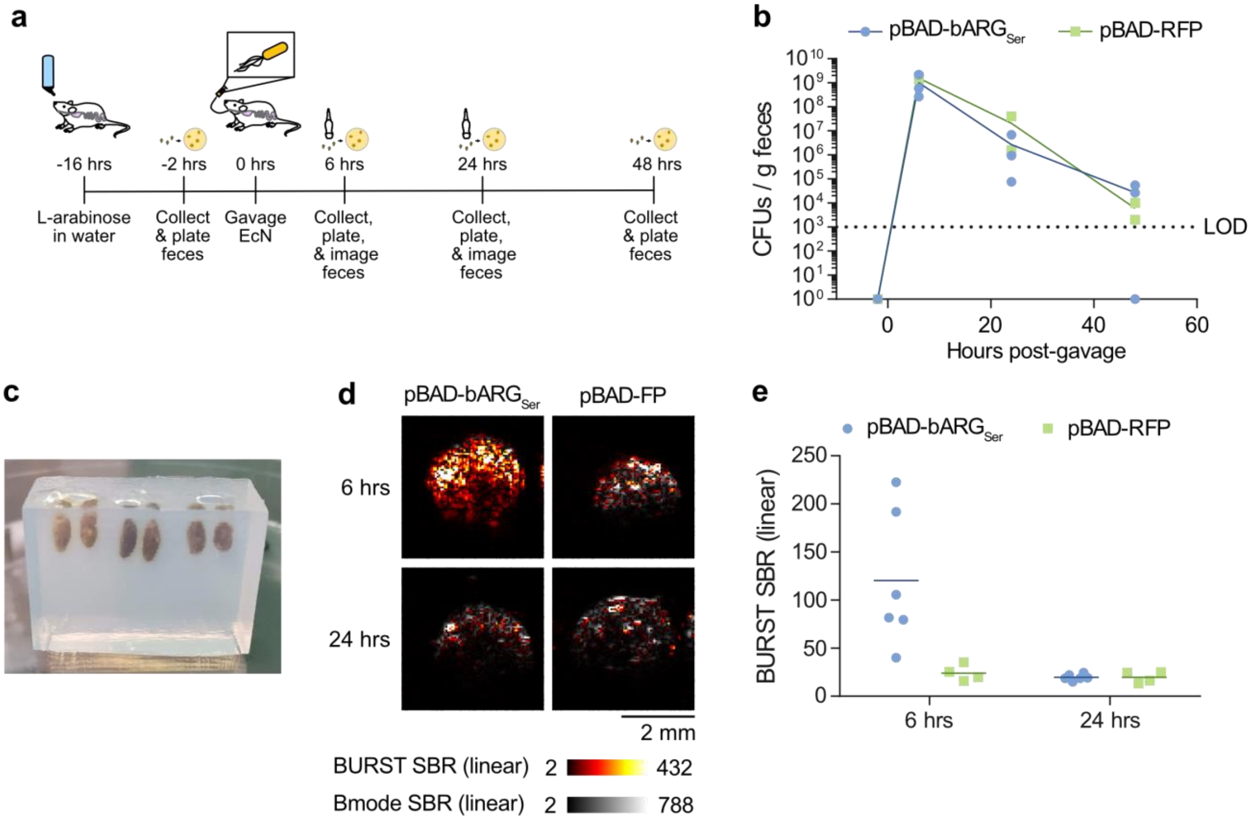

Figure S10: Ultrasound imaging of bARG$_{Ser}$-expressing EcN in feces without antibiotic treatment. (**a**) Experimental design for testing L-arabinose-inducible bARG$_{Ser}$ expression in EcN in vivo without antibiotics. Mice were given water containing L-arabinose for 16 hours, and EcN containing pBAD-bARG$_{Ser}$ or the control plasmid pBAD-RFP were orally administered. Feces were collected at various time points and imaged with ultrasound and/or plated on selective media to measure colonization. (**b**) Colony forming units (CFUs) per gram of feces collected 2 hours before, and 6, 24, and 48 hours after oral gavage of the EcN strains. Limit of detection (LOD) was 1.7 x 10^3 CFU/g feces. Points represent biological replicates (N = 3 mice per strain) and lines connect the means. (**c-e**) Representative phantom containing feces for ultrasound imaging (c), representative ultrasound images of feces overlaying the thresholded BURST image (hot scale) over the B-mode image (grayscale) (d), and quantification of the BURST signal-to-background ratio (SBR) of feces (e). Points represent biological replicates (two fecal pellets were imaged per mouse, giving N = 6 per strain) and lines represent the mean.

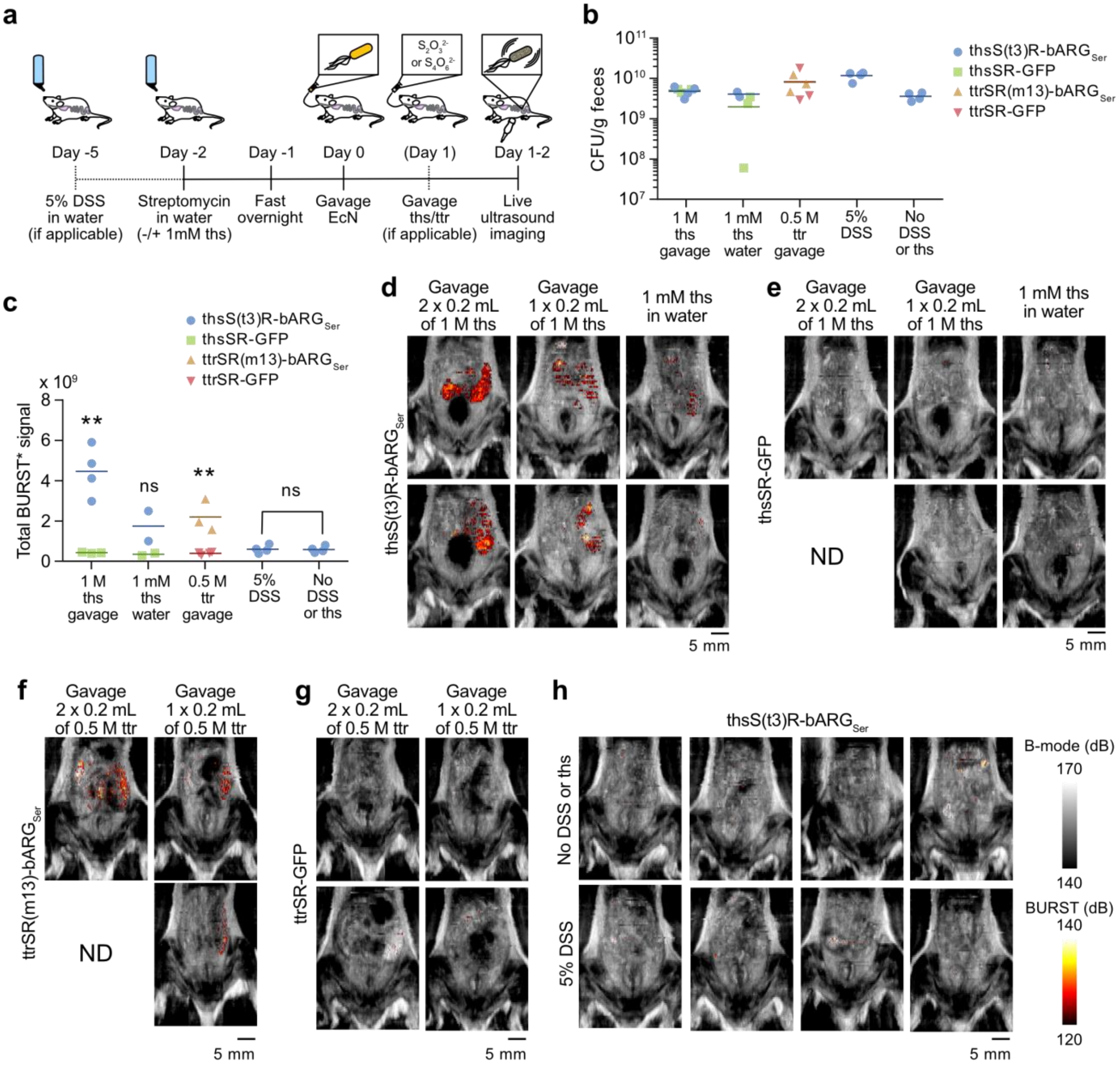

Figure S11: All replicates and additional data for testing thS(t3)R-bARG$_{Ser}$ and ttrSR(m13)-bARG$_{Ser}$ sensor EcN strains in vivo. (**a**) Diagram summarizing the experimental design involving DSS treatment, thiosulfate in the drinking water, or thiosulfate/tetrathionate administered via oral gavage. All mice received streptomycin in the drinking water for 2 days before oral gavage of sensor EcN strains, and all mice were imaged using the setup depicted in Fig. 8a one to two days after EcN gavage. (**b**) Colony forming units (CFU) per gram of feces one day after oral gavage of the sensor EcN strains. (**c**) Total BURST* ultrasound signal imaged in mice one day (1 mM ths water, 5% DSS, and no DSS or ths) or two days (1 M ths gavage and 0.5 M ttr gavage) after EcN gavage. (**d-e**) Overlay of the integrated BURST* images onto the integrated B-mode images over the depth for all mice colonized by thsS(t3)R-bARG$_{Ser}$ (d) or thsSR-GFP (e) EcN and treated with thiosulfate via oral gavage or in the drinking water. (**f-g**) Overlay of the integrated BURST* images onto the integrated B-mode images over the depth for all mice colonized by ttrSR(m13)-bARG$_{Ser}$ (f) or ttrSR-GFP (g) EcN and treated with tetrathionate via oral gavage. (**h**) Overlay of the integrated BURST* images onto the integrated B-mode images over the depth for all mice colonized by thS(t3)R-bARG$_{Ser}$ EcN and treated with DSS or left untreated (no DSS or thiosulfate).For (b) and (c), points represent biological

replicates (each replicate's ultrasound image is shown in d-f), lines represent the mean, and asterisks represent statistical significance by unpaired Student's t-tests (** = $p<0.01$, ns = no significance).

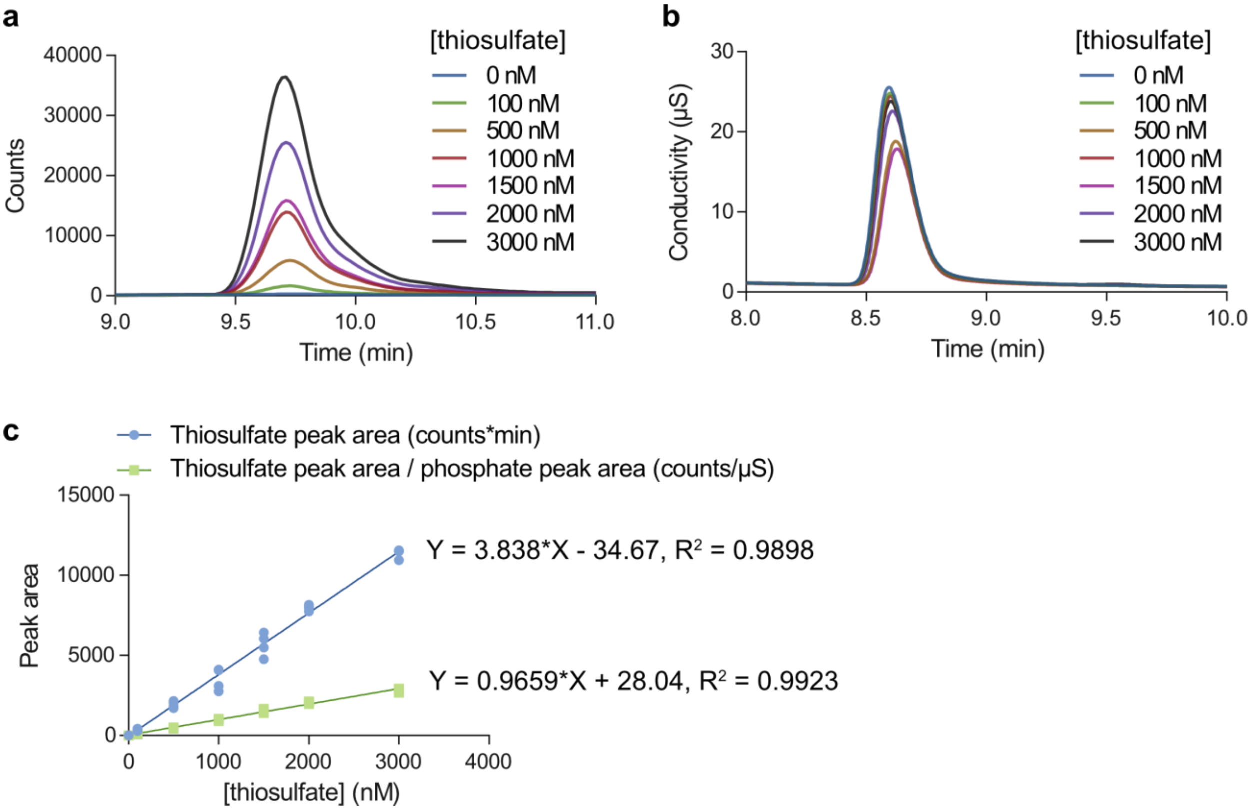

Figure S12: Ion chromatography-mass spectrometry (IC-MS) chromatograms and standard curves for quantifying thiosulfate. (**a**) Representative extracted ion chromatograms showing the thiosulfate peaks (m/z = 112.5-113.5 filter) in thiosulfate samples at concentrations ranging from 0 to 3000 nM in a PBS background. (**b**) Representative conductivity chromatograms showing the phosphate peaks in thiosulfate samples at concentrations ranging from 0 to 3000 nM in a PBS background. The concentration of PBS was the same in all samples, so the phosphate served as an internal standard to correct for variations in the injection volume. (**c**) Standard curve for the raw thiosulfate peak area and for the thiosulfate peak area normalized by the corresponding phosphate peak area. The normalized peak areas were used for quantification of thiosulfate in fecal and intestinal samples because normalization resulted in less variation between technical replicates. Points represent technical replicates (N=4) and lines represent linear regressions.

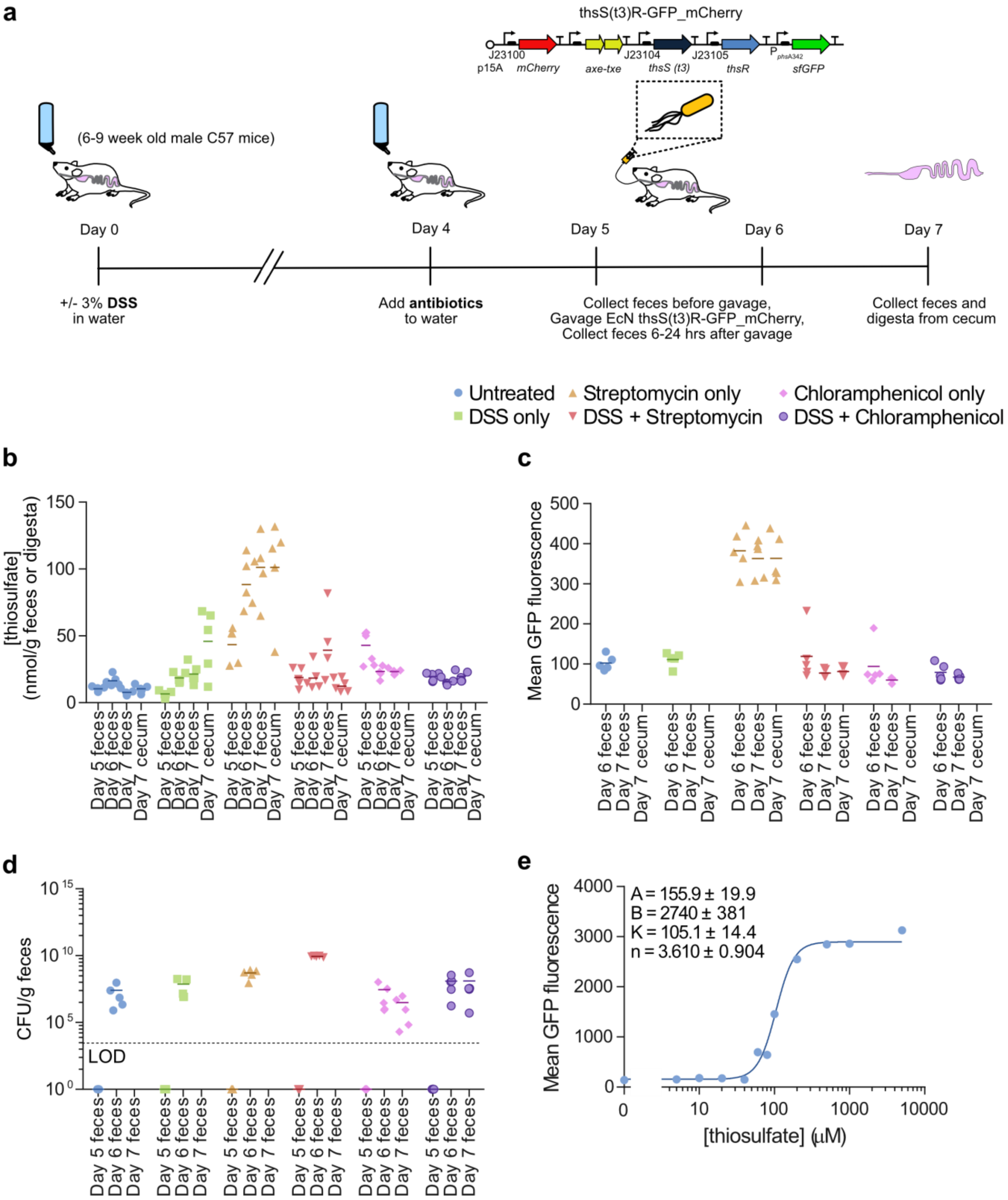

Figure S13: Measuring thiosulfate levels and thsS(t3)R-GFP_mCherry sensor activation in response to DSS and antibiotics in vivo. (**a**) Experimental design for testing the effect of DSS and antibiotics on intestinal thiosulfate levels and thiosulfate sensor activation. Mice were given water with 3% DSS or without DSS on day 0, and on day 4 antibiotics were added to the water. The thiosulfate-sensing EcN strain with plasmid thsS(t3)R-GFP_mCherry was administered via oral gavage on day 5 for antibiotic-treated mice and on day 6 for mice not given antibiotics. Feces were collected on days 5-7, cecal contents were collected on day 7, and they were analyzed via IC-MS, flow cytometry, and plating. (**b**) Concentrations of thiosulfate

measured via IC-MS in the feces or digesta of the cecum. (**c**) Mean GFP fluorescence of positive mCherry events of the sensor strain measured via flow cytometry. There is no flow cytometry data for mice not treated with antibiotic on day 7 because the strain did not colonize without antibiotics. (**d**) Colony forming units (CFU) per gram of feces measured by plating on selective media to assess colonization of the sensor bacteria. (**e**) In vitro characterization of the thsS(t3)R-GFP_mCherry EcN sensor strain in terms of mean GFP fluorescence measured via flow cytometry after inducing with varying thiosulfate concentrations in liquid culture at 37°C. Maximal sensor activation observed in vivo in streptomycin-treated mice was only 11.8% that of maximal sensor activation observed in vitro. Points represent biological replicates (N=5 for b-d, N=1 for e) and horizontal lines represent the mean. For (e), the curve represents a fit to the Hill equation (see Table S2) with parameters ± the standard error displayed on the upper left.

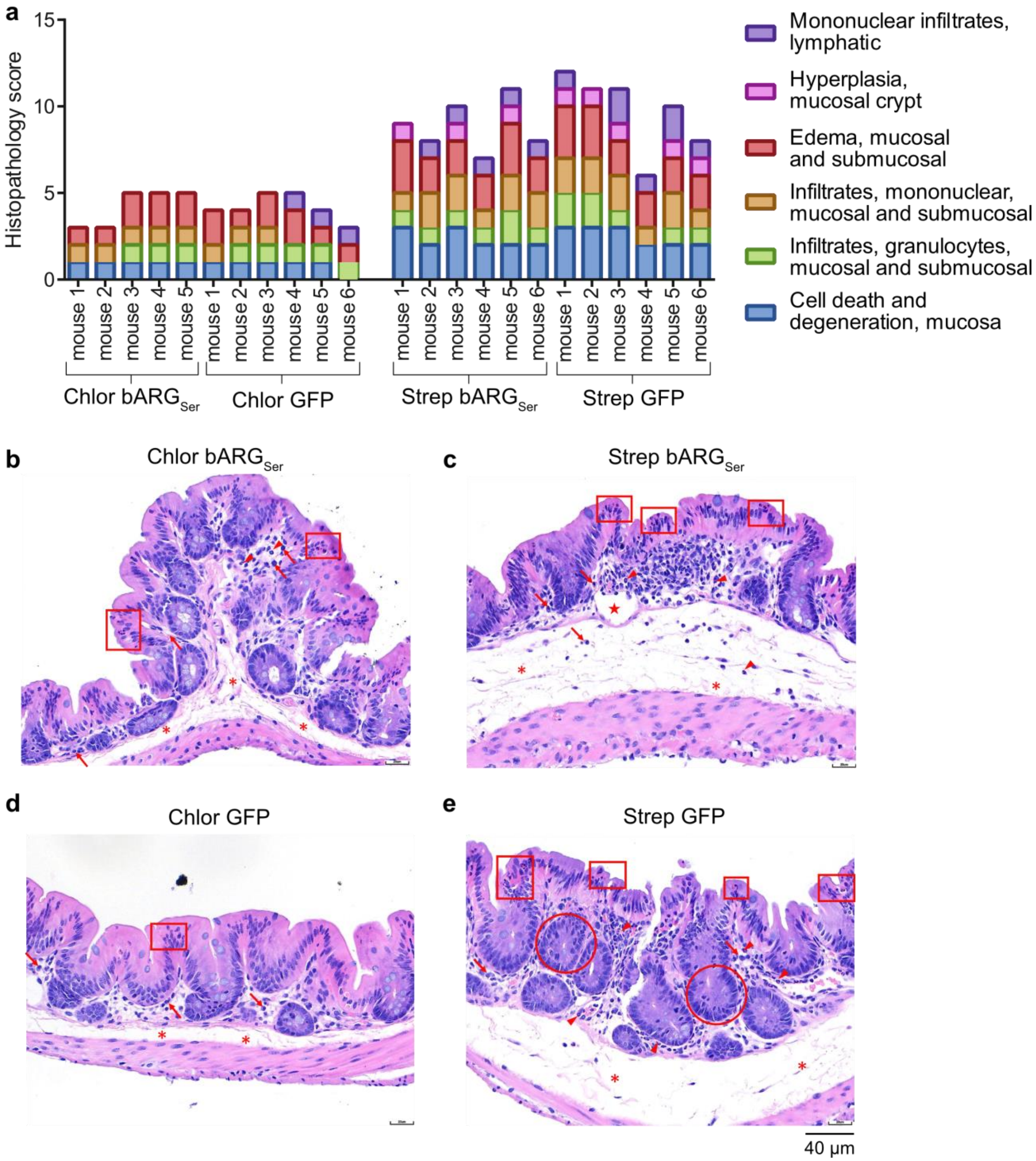

Figure S14: Full scoring data and additional histopathology images of cecal tissues from chloramphenicol- and streptomycin-treated mice. (**a**) Histopathology scoring of cecal tissues from chloramphenicol- and streptomycin-treated mice on day 5 of treatment broken down by category of abnormality. These data were aggregated for display in Fig. 5d. (**b-e**) Representative images of H&E-stained sections of cecal tissue on day 5 of antibiotic treatment for mice treated with chloramphenicol and colonized with thsS(t3)R-Bxb1_P7-bARG$_{Ser}$ EcN (b), treated with streptomycin and colonized with thsS(t3)R-Bxb1_P7-bARG$_{Ser}$ EcN (c), treated with chloramphenicol and colonized with thsS(t3)R-Bxb1_P7-GFP_mCherry EcN (d), and treated with streptomycin and colonized with thsS(t3)R-Bxb1_P7-GFP_mCherry EcN (e). Abnormalities are indicated in red: mucosal epithelial cell death and degeneration

(box), mucosal crypt hyperplasia (circle), mucosal/submucosal edema (asterisk), mononuclear infiltrates (arrow), granulocytic infiltrates (arrowhead), dilated lymphatic (star).

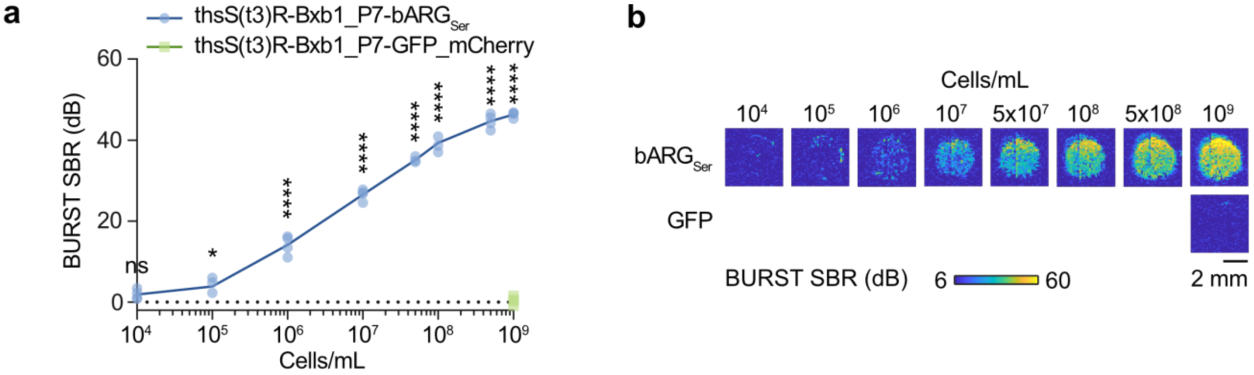

Figure S15: Ultrasound detection limit of optimized thiosulfate sensor. (**a-b**) BURST signal-to-background ratio (SBR) (a) and representative images (b) of varying cell concentrations of thsS(t3)R-Bxb1_P7-bARG$_{Ser}$ and thsS(t3)R-Bxb1_P7-GFP_mCherry induced with 90 µM thiosulfate. Asterisks represent statistical significance by two-tailed, unpaired Student's t-tests (ns = no significance, * = $p < 0.05$, **** = $p < 0.0001$; p-values from left to right: 1.018E-01, 1.743E-02, 3.950E-05, 8.049E-08, 1.497E-09, 2.424E-08, 1.015E-08, 3.501E-10). Points represent biological replicates (N=4), which are each the mean of two technical replicates, and curves represent the mean. Strains were grown on plates at 37°C for 20-24 hours, suspended in PBS, and cast in agarose phantoms at varying concentrations.

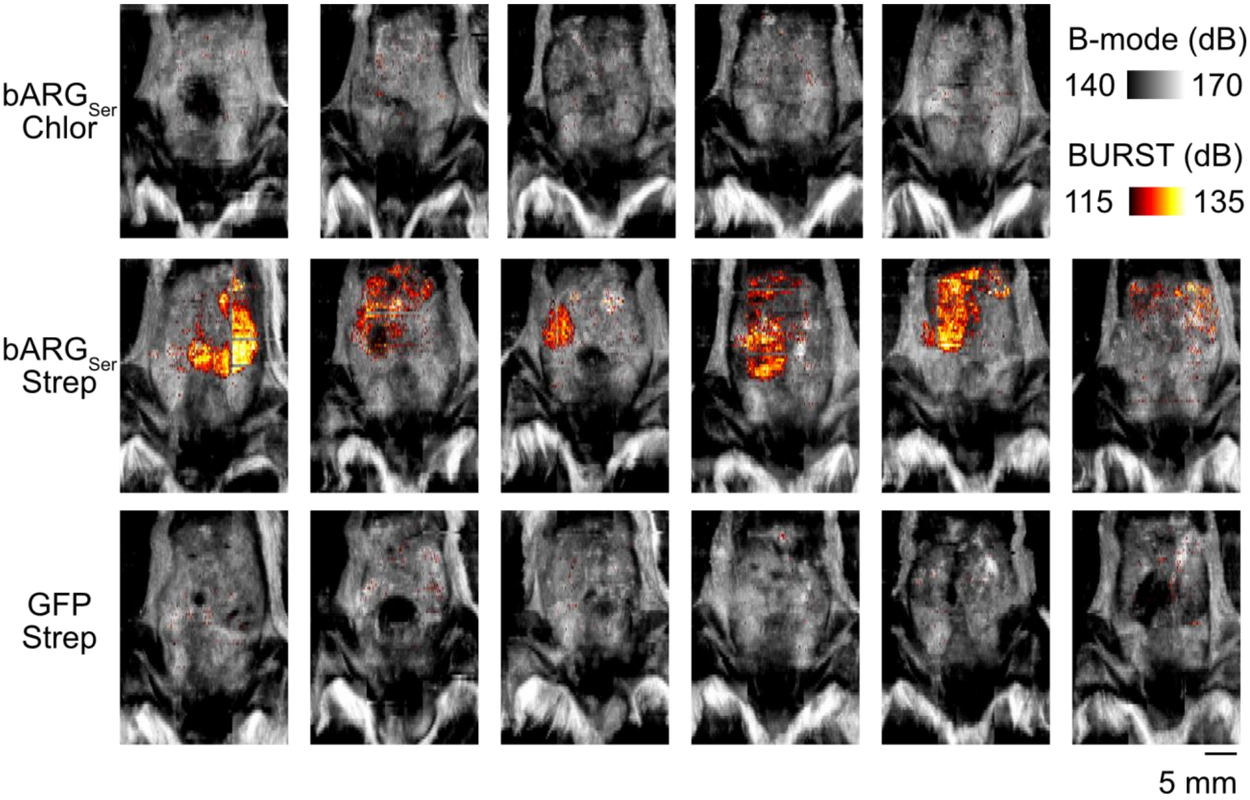

Figure S16: All ultrasound images of mice from the experiment depicted in Fig. 5. The integrated BURST* signal was overlaid onto the integrated B-mode signal for all mice treated with chloramphenicol and colonized with thsS(t3)R-Bxb1_P7-bARG$_{Ser}$ EcN (bARG$_{Ser}$ Chlor), for all mice treated with streptomycin and colonized with thsS(t3)R-Bxb1_P7-bARG$_{Ser}$ EcN (bARG$_{Ser}$ Strep), and for all mice treated with streptomycin and colonized with thsS(t3)R-Bxb1_P7-GFP_mCherry EcN (GFP Strep) on day 3.

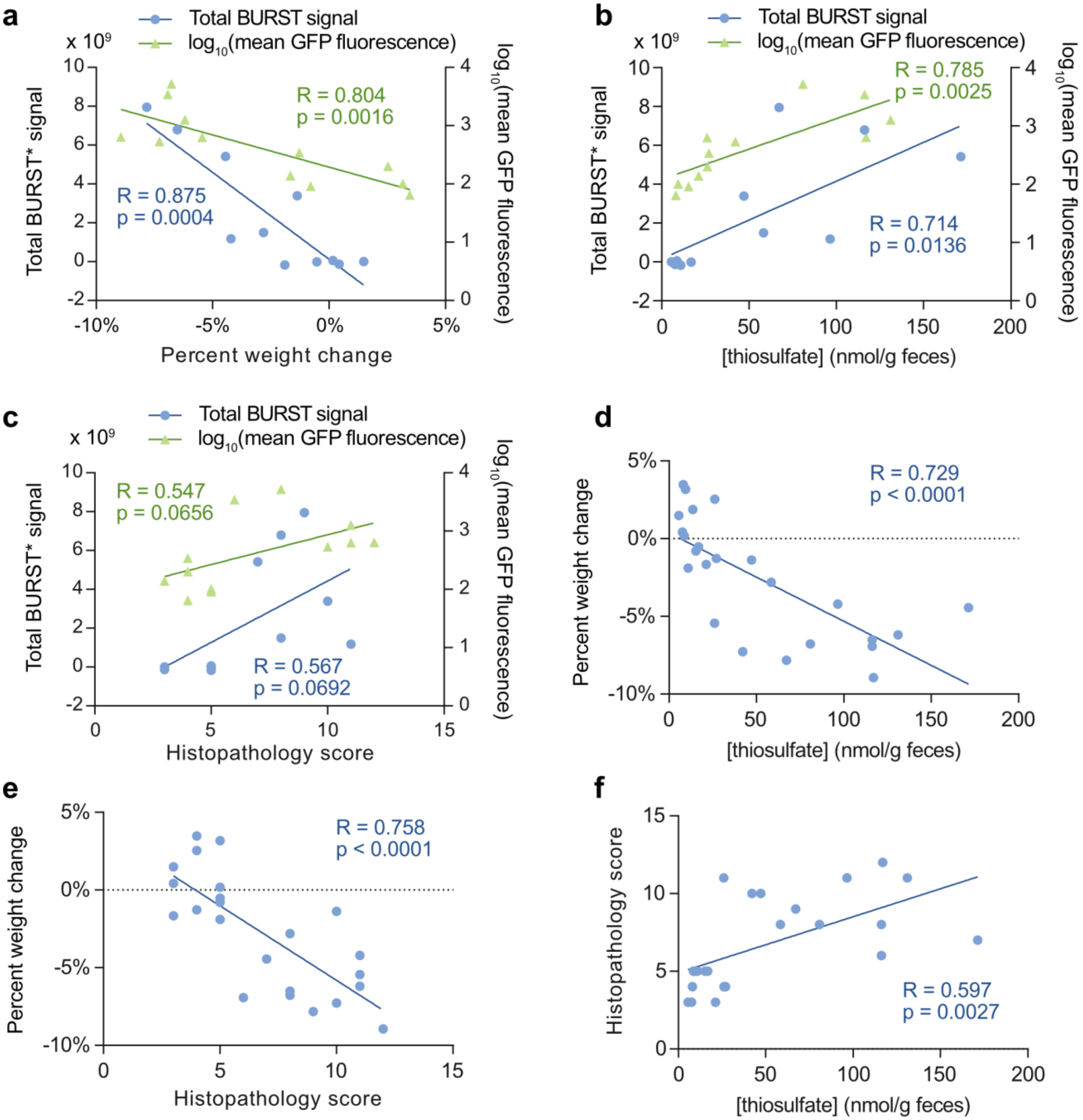

Figure S17: Correlations between thiosulfate sensor activation and disease severity in chloramphenicol- and streptomycin-treated mice. (**a-c**) Total BURST* signal imaged on day 3 or mean GFP fluorescence measured on day 4 versus the percent weight change on day 2 before mice were fasted with tail cups (a), versus the fecal thiosulfate levels measured via IC-MS on day 4 (b), and versus the histopathology score of cecal tissue on day 5 (c). (**d-e**) percent weight change on day 2 before mice were fasted with tail cups versus the fecal thiosulfate levels measured via IC-MS on day 4 (d), and versus the histopathology score of cecal tissue on day 5 (e). (**f**) Histopathology score of cecal tissue on day 5 versus the fecal thiosulfate levels measured via IC-MS on day 4. Points represent biological replicates, lines represent linear regressions where R represents the goodness of fit ($R = \sqrt{1 - \frac{sum\text{−}of\text{−}squares\ of\ distances\ from\ regression\ line}{sum\text{−}of\text{−}squares\ of\ distances\ from\ null\ hypothesis\ line}}$), and p values indicate whether the slope is significantly non-zero.

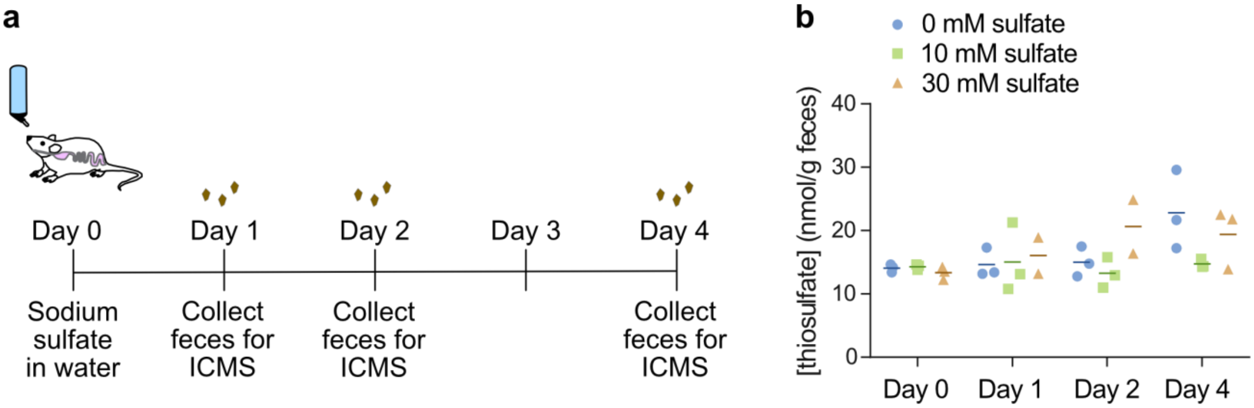

Figure S18: Effect of sodium sulfate in drinking water on fecal thiosulfate levels. (**a**) Experimental design, where mice were given drinking water containing 0, 10, or 30 mM sodium thiosulfate on day 0, and feces were collected and analyzed via IC-MS on days 0, 1, 2, and 4. 10 mM sodium thiosulfate corresponds to the concentration of sulfate in 5 g/L streptomycin sulfate. (**b**) Concentration of fecal thiosulfate measured by IC-MS. Supplementing the drinking water with sulfate did not significantly affect the fecal thiosulfate levels at any time point. Points represent biological replicates (N = 3 mice) and lines represent the mean.

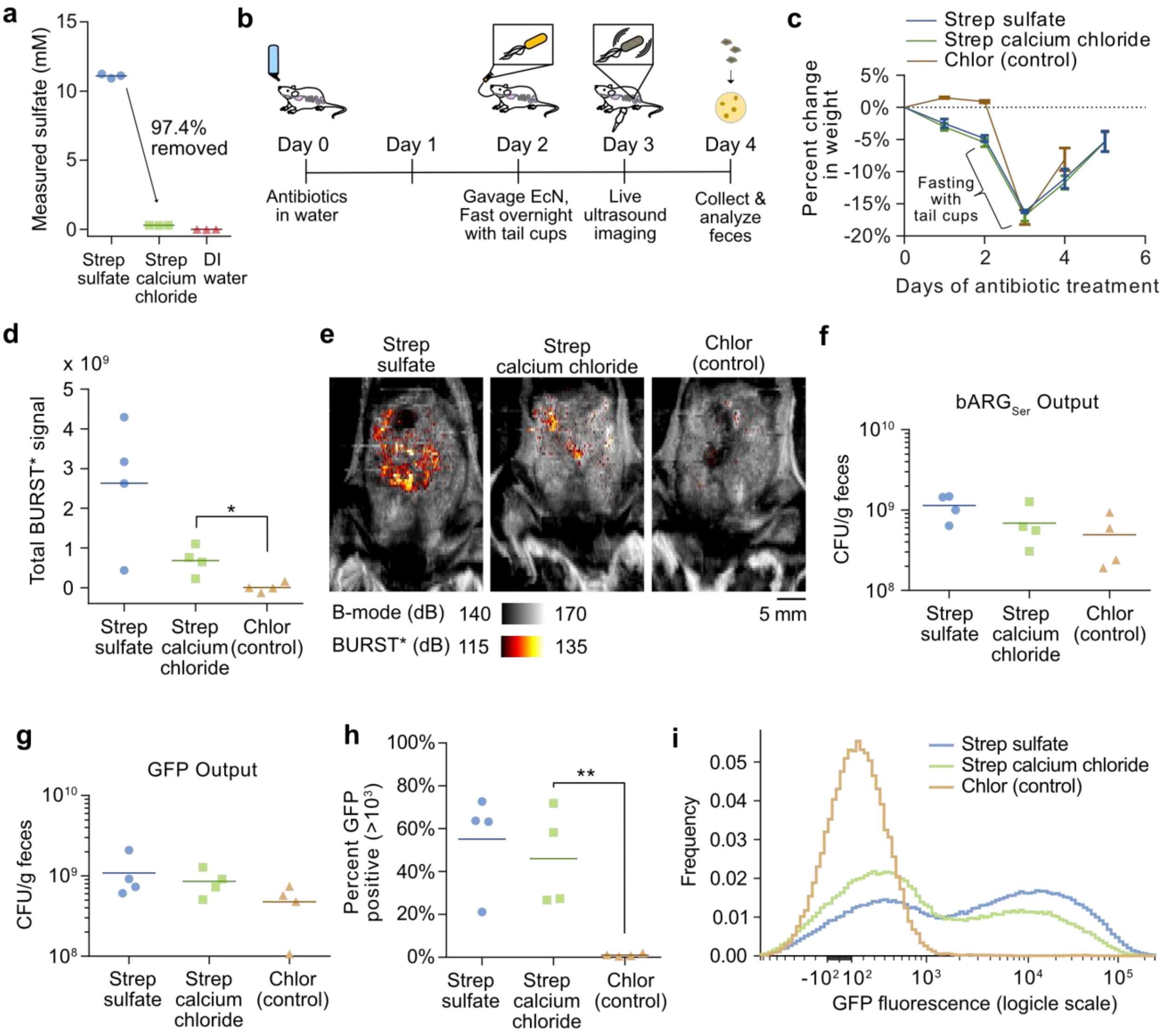

Figure S19: Effect of sulfate removal from streptomycin sulfate on this model of inflammatory dysbiosis. (**a**) Measured sulfate concentration of solutions of streptomycin sulfate, streptomycin calcium chloride (where calcium chloride precipitation was used to remove the sulfate, see methods), and the DI water used to prepare these solutions. Points represent technical replicates and lines represent means. (**b**) Experimental design for testing EcN strains containing plasmids for the optimized integrase-based switch thiosulfate sensors, thsS(t3)R-Bxb1_P7-bARG$_{Ser}$ or thsS(t3)R-Bxb1_P7-GFP_mCherry, in mice treated with various antibiotic solutions. (**c**) Percent change in weight after addition of streptomycin sulfate, streptomycin calcium chloride, or chloramphenicol to the drinking water. Lines connect the means and error bars represent the standard error of the mean (N = 4). (**d-e**) Total BURST* signal (d) and representative ultrasound images (e) overlaying the integrated BURST* signal onto the integrated B-mode signal for mice colonized with thsS(t3)R-Bxb1_P7-bARG$_{Ser}$ EcN and treated with streptomycin sulfate, streptomycin calcium chloride, or chloramphenicol. P-value: 0.0117117 (d). (**f-g**) Colony forming units (CFU) per gram of feces on day 4 for mice treated with streptomycin sulfate, streptomycin calcium chloride, or chloramphenicol and colonized with thsS(t3)R-Bxb1_P7-bARG$_{Ser}$ EcN (f) or thsS(t3)R-Bxb1_P7-GFP_mCherry EcN (g). (**h-i**) Percent GFP positive events (> 10^3) (h) and aggregate histograms of GFP fluorescence (i) from flow cytometry analysis of feces from mice colonized with thsS(t3)R-Bxb1_P7-GFP_mCherry EcN on day 4. P-value: 0.00737297 (h). Asterisks represent statistical significance by two-tailed, unpaired Student's t-tests

(* = $p < 0.05$, ** = $p < 0.01$). For (d), (f), (g), and (h), points represent biological replicates (N=4) and lines represent means

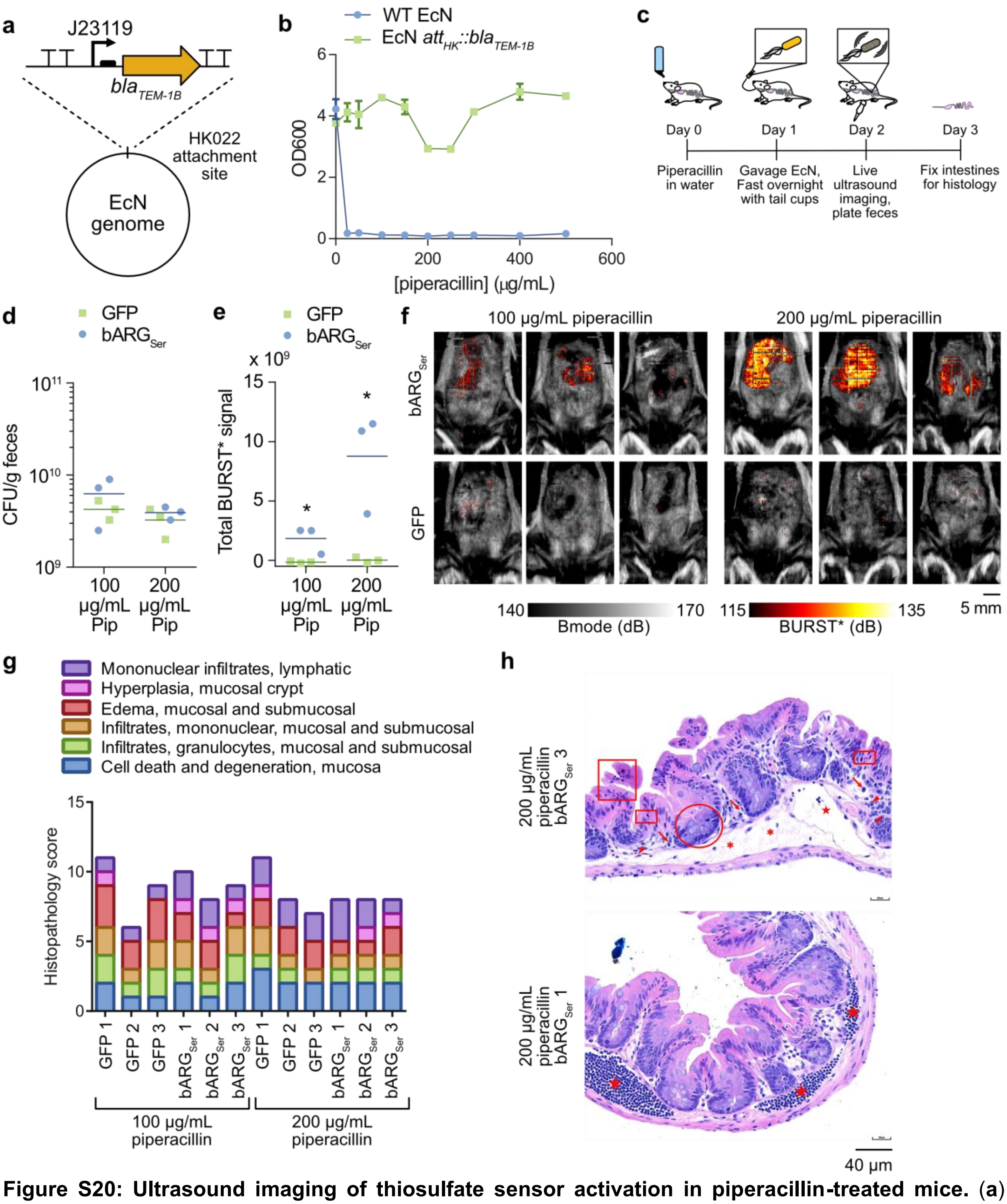

Figure S20: Ultrasound imaging of thiosulfate sensor activation in piperacillin-treated mice. (**a**) Diagram of genomic modification of EcN to confer piperacillin resistance. The beta lactamase gene *bla_{TEM-1B}* known to confer piperacillin resistance in *E. coli*[4,5] was placed under control of the strong constitutive promoter J23119. This cassette was integrated into the genome at the phage HK022 attachment site in EcN using the clontegration system[6]. See Table S3 for sequencing confirmation. (**b**) Optical density at 600 nm (OD600) after incubating wild-type (WT) EcN and the att_{HK}:bla_{TEM-1B} EcN in media at varying piperacillin

concentrations to confirm piperacillin resistance of the att_{HK}:bla_{TEM-1B} strain. Points represent the mean of two biological replicates, error bars represent the standard deviation, and lines connect the points. (**c**) Experimental design for testing EcN strains containing plasmids for the optimized integrase-based switch thiosulfate sensors, thsS(t3)R-Bxb1_P7-$bARG_{Ser}$ or thsS(t3)R-Bxb1_P7-GFP_mCherry, in piperacillin-treated mice. One day after piperacillin was administered via drinking water, the EcN strains were administered via oral gavage and the next day mice were scanned with ultrasound using the setup depicted in Fig S8a. One day later on day 3, mice were sacrificed and their intestines were fixed for histology. (**d-e**) Colony forming units (CFU) per gram of feces (d) and total BURST* ultrasound signal imaged (e) on day 2 of piperacillin treatment (100 or 200 μg/mL) for mice colonized by thsS(t3)R-Bxb1_P7-$bARG_{Ser}$ ($bARG_{Ser}$) or thsS(t3)R-Bxb1_P7-GFP_mCherry (GFP) EcN. Points represent biological replicates, lines represent the mean, and asterisks represent statistical significance by two-tailed, unpaired Student's t-tests (* = $p < 0.05$). (**f**) Ultrasound images overlaying the integrated BURST* signal onto the integrated B-mode images for all mice on day 2 of piperacillin treatment. (**g**) Histopathology scoring of cecal tissues from piperacillin-treated mice on day 3 of treatment broken down by category of abnormality. Mice which received 200 μg/mL piperacillin did not exhibit significantly more signs of disease than mice which received 100 μg/mL piperacillin, but both piperacillin-treated groups exhibited more signs of disease than chloramphenicol-treated mice (see Fig. S14a). (**h**) Representative images of H&E-stained sections of cecal tissue on day 3 of piperacillin treatment. Abnormalities are indicated in red: mucosal epithelial cell death and degeneration (box), mucosal crypt hyperplasia (circle), mucosal/submucosal edema (asterisk), mononuclear infiltrates (arrow), granulocytic infiltrates (arrowhead), dilated lymphatic (star).

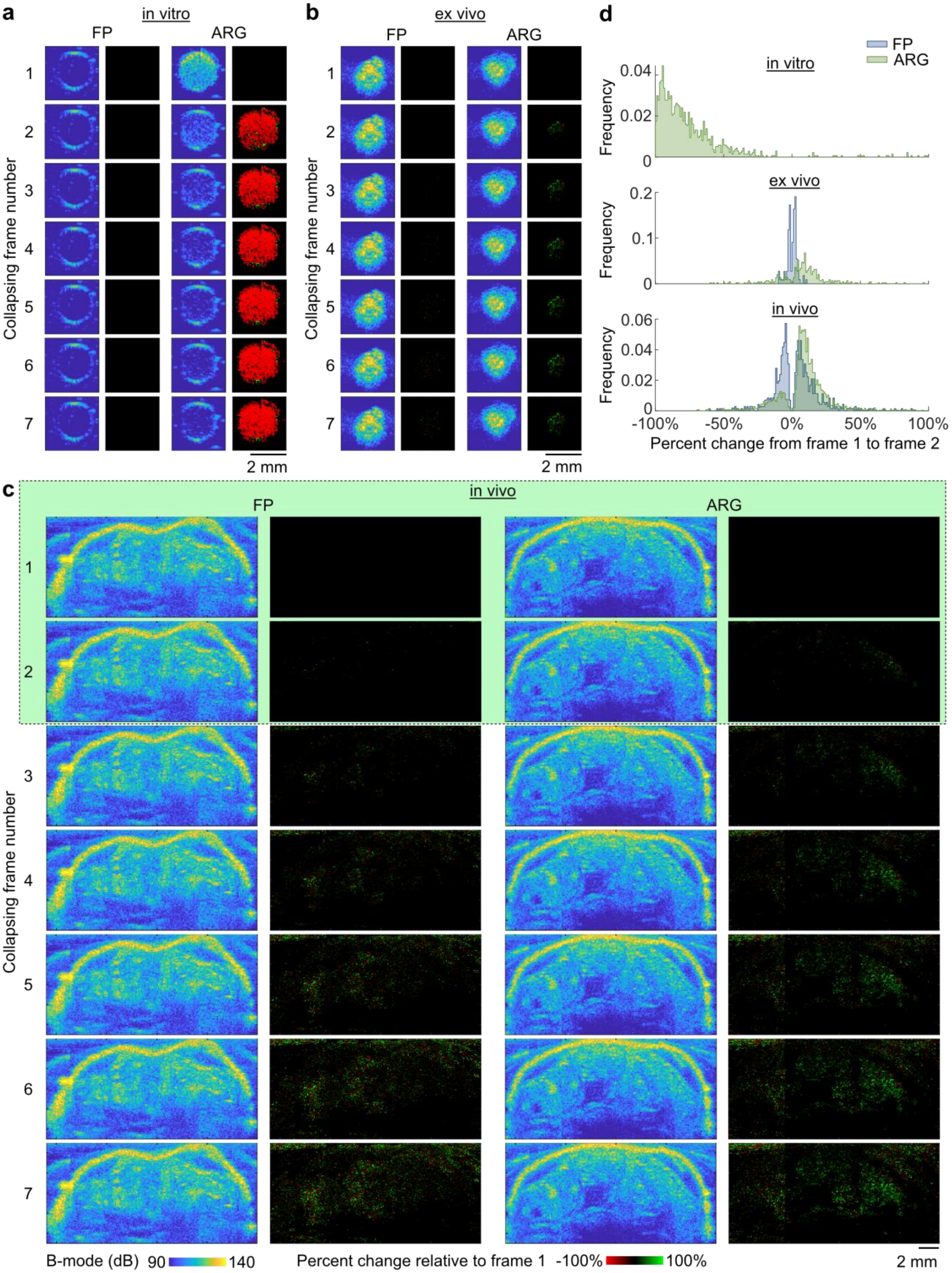
a
in vitro
FP
ARG
Collapsing frame number
1
2
3
4
5
6
7
2 mm
b
ex vivo
FP
ARG
Collapsing frame number
1
2
3
4
5
6
7
2 mm
d
FP
ARG
in vitro
Frequency
0.04
0.02
0
ex vivo
Frequency
0.2
0.1
0
in vivo
Frequency
0.06
0.04
0.02
0
-100%
-50%
0%
50%
100%
Percent change from frame 1 to frame 2
c
in vivo
FP
ARG
Collapsing frame number
1
2
3
4
5
6
7
B-mode (dB) 90
140
Percent change relative to frame 1 -100%
100%
2 mm

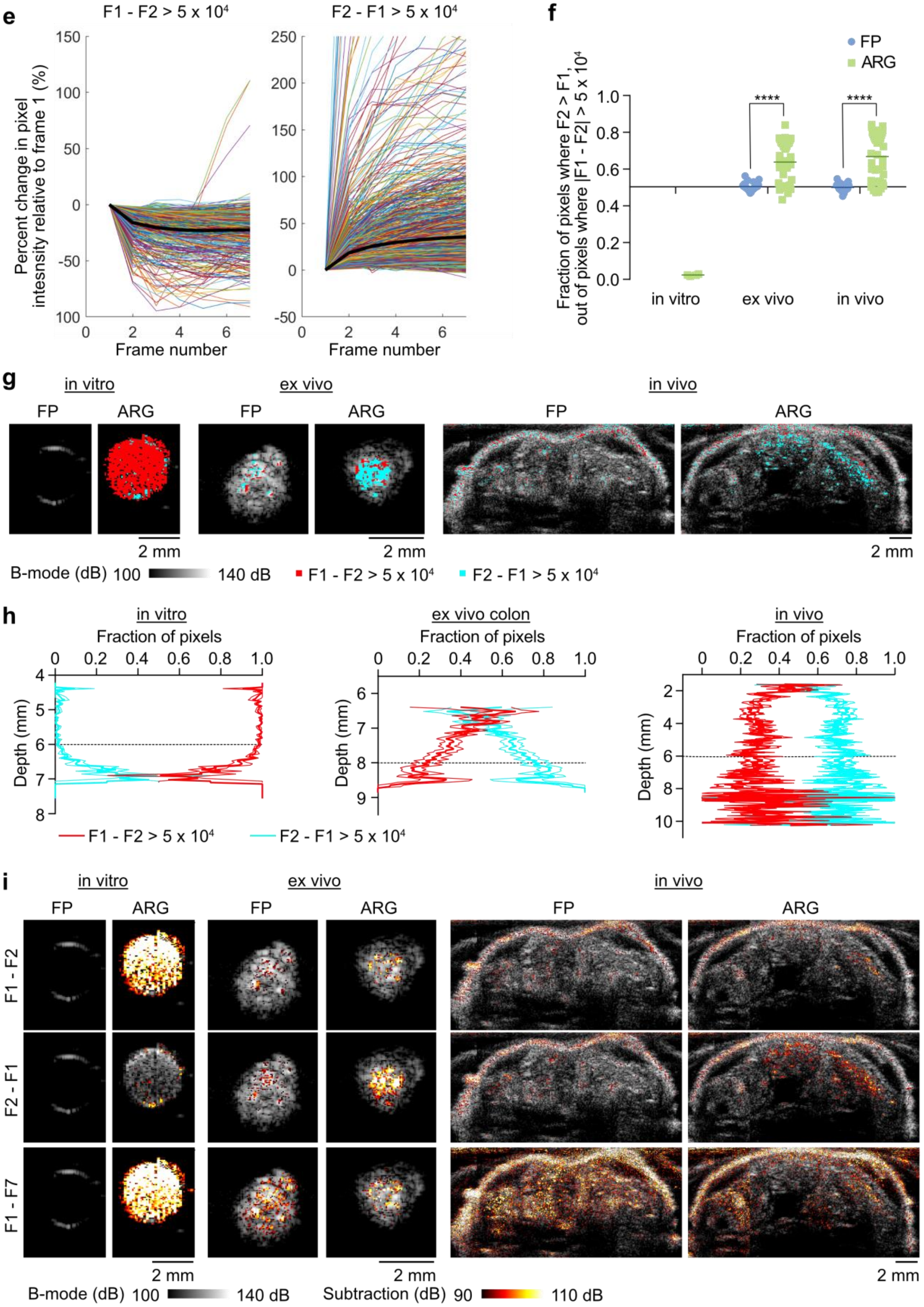

e
F1 - F2 > 5 x 10⁴
F2 - F1 > 5 x 10⁴
Percent change in pixel intesnity relative to frame 1 (%)
Frame number
f
FP
ARG
Fraction of pixels where F2 > F1, out of pixels where |F1 - F2| > 5 x 10⁴
****
in vitro
ex vivo
in vivo
g
in vitro
ex vivo
in vivo
FP
ARG
2 mm
B-mode (dB) 100
140 dB
F1 - F2 > 5 x 10⁴
F2 - F1 > 5 x 10⁴
h
in vitro
ex vivo colon
in vivo
Fraction of pixels
Depth (mm)
F1 - F2 > 5 x 10⁴
F2 - F1 > 5 x 10⁴
i
in vitro
ex vivo
in vivo
FP
ARG
F1 - F2
F2 - F1
F1 - F7
2 mm
B-mode (dB) 100
140 dB
Subtraction (dB) 90
110 dB

Figure S21: Comparison of processing BURST images in vitro, ex vivo, and in vivo. (**a-c**) B-mode images of the 7 collapsing frames (left) and percent difference images relative to the first collapsing frame (right) acquired using a rapid BURST script (that uses 3 focused beams at a time) of representative in vitro samples of EcN expressing $bARG_{Ser}$ or a fluorescent protein (FP) at 5 x 10^8 cells/mL (a), ex vivo colons colonized by $bARG_{Ser}$ or FP-expressing EcN (b), and in vivo intestines of mice colonized by $bARG_{Ser}$ or FP-expressing EcN (c). Color bars are the same for (a-c). To exclude noise, for the percent difference images, only pixels that had an absolute difference of 5 x 10^4 or greater relative to the first collapsing frame were included. For (c), only the difference between the first two collapsing frames (green box) is useful due to tissue motion. (**d**) Histograms of the percent changes in pixel intensity from the first collapsing frame (frame 1) to the second collapsing frame (frame 2) for the representative in vitro, ex vivo, and in vivo images from (a-c). Only pixels that had an absolute difference of 5 x 10^4 or greater were included; the FP in vitro images did not have any of these pixels. (**e**) Example pixel traces in terms of percent difference relative to frame 1 over the seven collapsing frames for the representative in vivo ARG acquisitions, categorized into pixels where the signal in the first collapsing frame (F1) was greater than the second (F2) by more than 5 x 10^4 (F1 - F2 > 5 x 10^4, left), and the pixels where the signal from the second collapsing frame (F2) was greater than the first (F1) by more than 5 x 10^4 (F2 - F1 > 5 x 10^4, right). Thin colored lines represent individual traces and bold black lines represent the mean. (**f**) Fraction of pixels where F2 > F1 out of all pixels that had an absolute difference of greater than 5 x 10^4 between frames 1 and 2 for representative in vivo, ex vivo, and in vivo samples containing FP- or $bARG_{Ser}$-expressing EcN. For in vitro samples, each point represents a biological replicate (N=4); FP in vitro acquisitions did not have any pixels that had an absolute difference of greater than 5 x 10^4 between frames 1 and 2 so no data points are shown for this category. For ex vivo and in vivo samples, each point represents a BURST acquisition at a different location in the same mouse/intestines where the number of pixels that had an absolute difference of greater than 5 x 10^4 between frames 1 and 2 was greater than 500 (from left to right, N = 28, 42, 15, 41). Lines represent the mean. Asterisks represent statistical significance by unpaired Student's t-tests (**** = $p < 0.00001$; p-values from left to right: 2.163525e-008, 4.295376e-006). (**g**) Representative overlay images of the pixels where F1-F2 > 5 x 10^4 (red), and the pixels where F2-F1 > 5 x 10^4 (cyan). (**h**) Fraction of pixels where F1-F2 > 5 x 10^4 or F2-F1 > 5 x 10^4 versus the depth from the transducer for representative in vitro samples, ex vivo colons, and in vivo intestines containing ARG-expressing EcN. Bold colored lines represent the mean and thin colored lines represent the standard error of the mean (N = 4 biological replicates for in vitro samples, N = 30 cross-sectional images of an ex vivo colon which each contained more than 200 pixels where |F1-F2| > 5 x 10^4, and N = 35 cross-sectional images of intestines in vivo which each contained more than 500 pixels where |F1-F2| > 5 x 10^4). Dashed black lines represent the transducer focus. (**i**) Representative overlay images of different subtraction BURST images (hot scale) onto the B-mode image (greyscale): the first collapsing frame minus the second (F1-F2), the second collapsing frame minus the first (F2-F1), and the first collapsing frame minus the last (F1-F7). The negative portion of the subtraction images were removed for conversion to dB, and then the subtraction images were thresholded at 90 dB for the overlay.

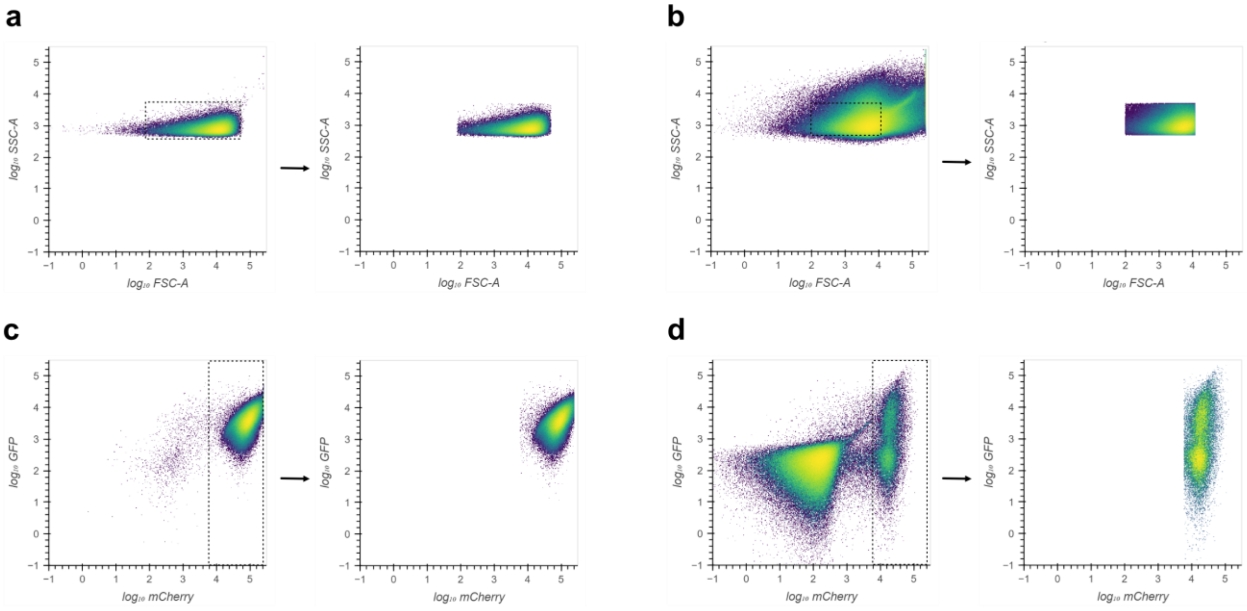

Figure S22: Flow cytometry gating strategy. (**a-b**) Representative SSC-A versus FSC-A plots showing that events were first gated on FSC-A and SSC-A characteristic of *E. coli* (dashed box) for both in vitro (a) and fecal (b) samples. (**c-d**) Representative GFP versus mCherry plots showing that events were then gated on positive mCherry fluorescence for both in vitro (c) and fecal (d) samples, as all fluorescent protein EcN strains constitutively expressed mCherry. These gates were applied and the resulting downstream analyses (GFP histograms, mean GFP fluorescence, and percent GFP positive cells) were performed using custom Python scripts. The gates in (a) and (c) were applied to all in vitro flow cytometry samples, which are depicted in Fig. 3e-f and Fig. S6i-j. The gates in (b) and (d) were applied to all fecal flow cytometry samples, which are depicted in Fig. 5j-l, Fig. S13c, e, Fig. S17a-c, and Fig. S19h-i.

Supplementary Note 1: **BURST* imaging.**
Ex vivo and in vivo imaging in our study was performed using BURST* imaging. A modification of conventional BURST was required to obtain robust signals specific to ARG expression in samples with high background due to GI tissue and luminal contents. For in vitro samples containing ARGs, the collapse of the gas vesicles was apparent from raw B-mode images acquired at the collapsing pressure of BURST (Fig. S21a). In contrast, for ex vivo and in vivo samples containing ARGs, the difference in pixel intensity over the collapsing frames was small relative to the background and was confounded by tissue motion in later frames (Fig. S21b-c). For example, pixels were distributed around -70% difference from frame 1 to frame 2 for a representative in vitro sample with ARGs, while pixels were distributed around -16% and +18% difference from frame 1 to frame 2 for a representative in vivo sample with ARGs (Fig. S21d). Furthermore, the pixel intensities mostly decreased for ARG-containing in vitro samples while the pixel intensities tended to increase for ARG-containing ex vivo and in vivo samples over successive collapsing frames (Fig. S21d-e). For pixels that displayed an absolute difference of 5×10^4 or greater, on average 98% of these pixels decreased from frame 1 to frame 2 for in vitro samples containing ARGs, while on average 64% and 67% of these pixels increased from frame 1 to frame 2 for ex vivo and in vivo acquisitions containing ARGs (Fig. S21f). This asymmetry and magnitude of changes is absent in control ex vivo and in vivo acquisitions with cells expressing fluorescent proteins (FP) (Fig. S21f). Thus, the ARG-specific signal in the ex vivo and in vivo acquisitions was characterized by a higher fraction of pixels with an increase in intensity from frame 1 to frame 2 of more than 5×10^4. This trend could at least partially be caused by shadowing, as the percentage of pixels where $F2-F1 > 5 \times 10^4$ tended to increase with the depth from the transducer (Fig. S21g-h), but further work is needed to investigate the mechanism behind this trend.

Based on these trends, we used the following subtraction-based methods to calculate BURST images. For in vitro samples, the last collapsing frame was subtracted from the first (F1-F7) because the first collapsing frame displayed the most signal while the last frame displayed the least signal, and there was no movement to confound the signal across the 7 frames (Fig. S21i). For ex vivo and in vivo acquisitions, the first collapsing frame was subtracted from the second (F2-F1) because the ARG-containing samples exhibited a high fraction of pixels with an increase in intensity from frame 1 to frame 2 whereas control samples did not (Fig. S21i), and analyzing temporally adjacent frames minimized tissue motion artifacts.

Supplementary Video Captions

Supplementary Video 1: BURST*/B-mode tomogram of an arabinose- and streptomycin-treated mouse colonized by pBAD-bARG$_{Ser}$ EcN

Supplementary Video 2: BURST*/B-mode tomogram of an arabinose- and streptomycin-treated mouse colonized by pBAD-RFP EcN

Supplementary Video 3: BURST*/B-mode tomogram of a chloramphenicol-treated mouse colonized by thsS(t3)R-Bxb1_P7-bARG$_{Ser}$ EcN

Supplementary Video 4: BURST*/B-mode tomogram of a streptomycin-treated mouse colonized by thsS(t3)R-Bxb1_P7-bARG$_{Ser}$ EcN

Supplementary Video 5: BURST*/B-mode tomogram of a streptomycin-treated mouse colonized by thsS(t3)R-Bxb1_P7-GFP_mCherry EcN

Supplementary References

1. Promoters/Catalog/Anderson - parts.igem.org. https://parts.igem.org/Promoters/Catalog/Anderson.
2. Abramson, J. *et al.* Accurate structure prediction of biomolecular interactions with AlphaFold 3. *Nature* 1–3 (2024) doi:10.1038/s41586-024-07487-w.
3. Daeffler, K. N. *et al.* Engineering bacterial thiosulfate and tetrathionate sensors for detecting gut inflammation. *Mol. Syst. Biol.* **13**, (2017).
4. Hubbard, A. T. M. *et al.* Piperacillin/tazobactam resistance in a clinical isolate of Escherichia coli due to IS26-mediated amplification of blaTEM-1B. *Nat. Commun.* **11**, 4915 (2020).
5. Hansen, K. H. *et al.* Resistance to piperacillin/tazobactam in Escherichia coli resulting from extensive IS26-associated gene amplification of blaTEM-1. *J. Antimicrob. Chemother.* **74**, 3179–3183 (2019).
6. St-Pierre, F. *et al.* One-Step Cloning and Chromosomal Integration of DNA. *ACS Synth. Biol.* **2**, 537–541 (2013).
