# Supplementary material for: Probiotic acoustic biosensors for noninvasive imaging of gut inflammation
Source: Nat Commun. 2025 Aug 25;16:7931. doi: 10.1038/s41467-025-62569-1 (PMC12379287; doi:10.1038/s41467-025-62569-1)
Supplement: Supplementary file 2 — Description of Additional Supplementary Files [file 41467_2025_62569_MOESM2_ESM.pdf]

Description of Additional Supplementary Files

Supplementary Video 1.gif (BURST*/B-mode tomogram of an arabinose- and streptomycin-treated mouse colonized by pBAD-bARG$_{Ser}$ EcN)

Supplementary Video 2.gif (BURST*/B-mode tomogram of an arabinose- and streptomycin-treated mouse colonized by pBAD-RFP EcN)

Supplementary Video 3.gif (BURST*/B-mode tomogram of a chloramphenicol-treated mouse colonized by thsS(t3)R-Bxb1_P7-bARG$_{Ser}$ EcN)

Supplementary Video 4.gif (BURST*/B-mode tomogram of a streptomycin-treated mouse colonized by thsS(t3)R-Bxb1_P7-bARG$_{Ser}$ EcN)

Supplementary Video 5.gif (Supplementary Video 5: BURST*/B-mode tomogram of a streptomycin-treated mouse colonized by thsS(t3)R-Bxb1_P7-GFP_mCherry EcN)

Supplementary Data 1: Primer sequences.xlsx
