# Supplementary figures and images for: Probiotic acoustic biosensors for noninvasive imaging of gut inflammation
Source: Nat Commun. 2025 Aug 25;16:7931. doi: 10.1038/s41467-025-62569-1 (PMC12379287; doi:10.1038/s41467-025-62569-1)

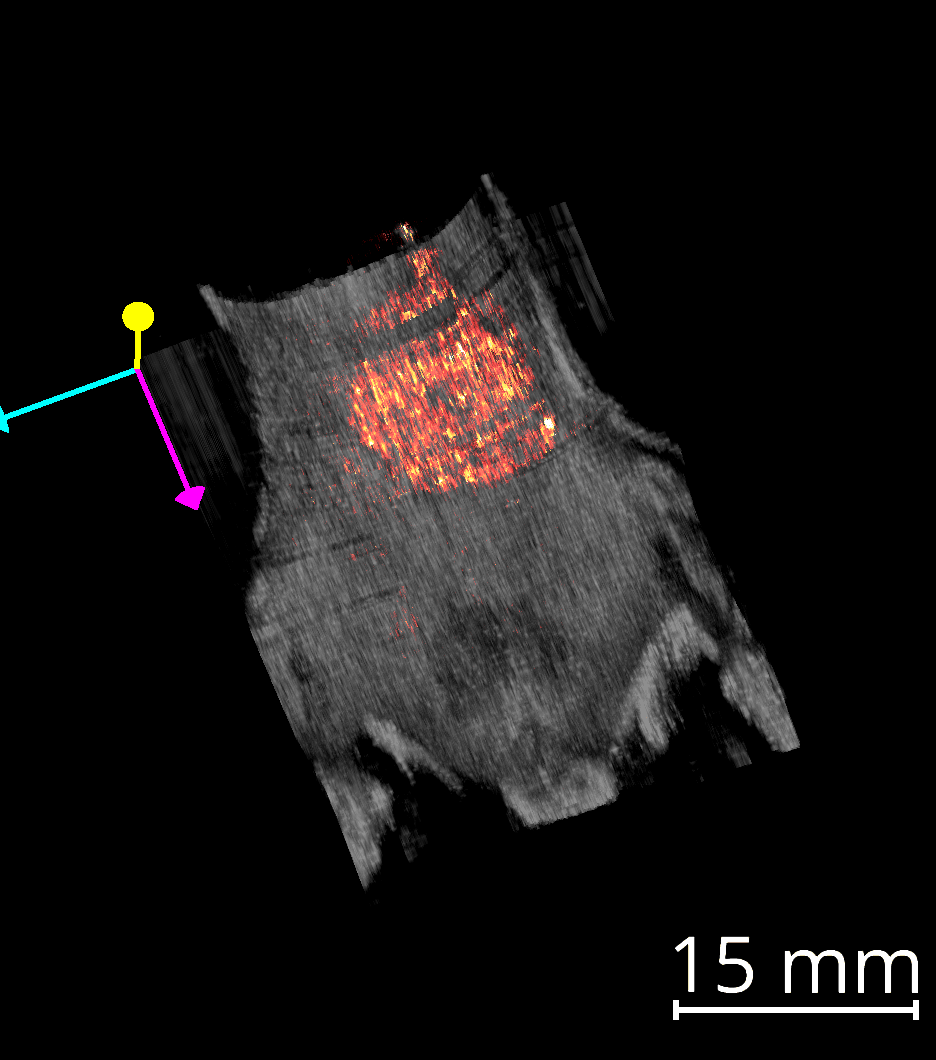

Supplement: Supplementary file 3 — Supplementary Video 1 [file 41467_2025_62569_MOESM3_ESM.gif]

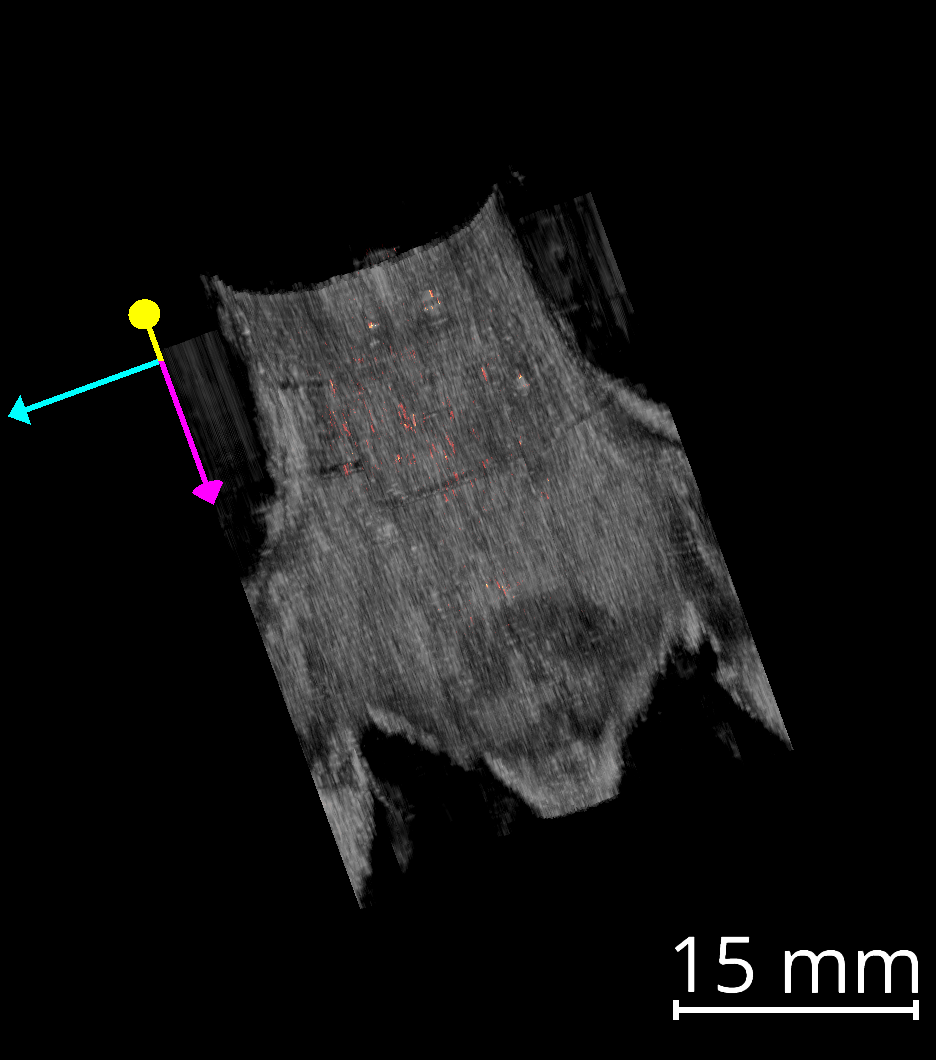

Supplement: Supplementary file 4 — Supplementary Video 2 [file 41467_2025_62569_MOESM4_ESM.gif]

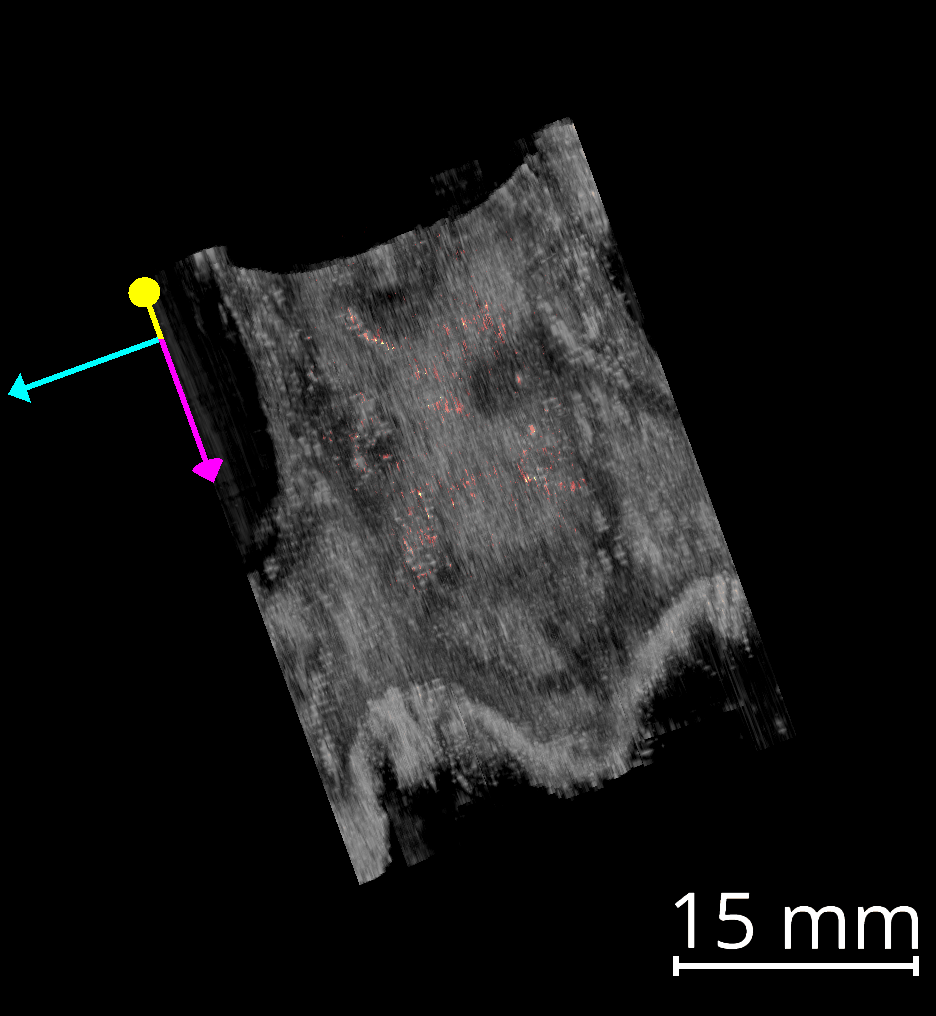

Supplement: Supplementary file 5 — Supplementary Video 3 [file 41467_2025_62569_MOESM5_ESM.gif]

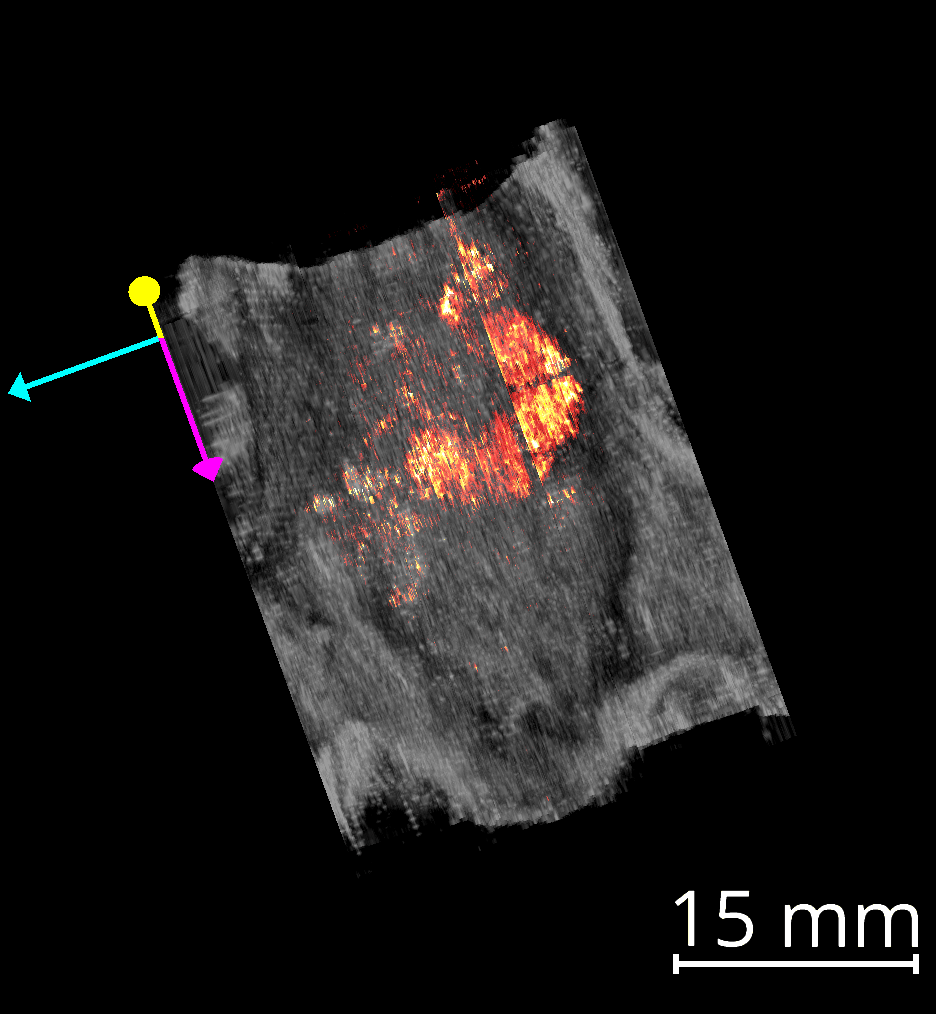

Supplement: Supplementary file 6 — Supplementary Video 4 [file 41467_2025_62569_MOESM6_ESM.gif]

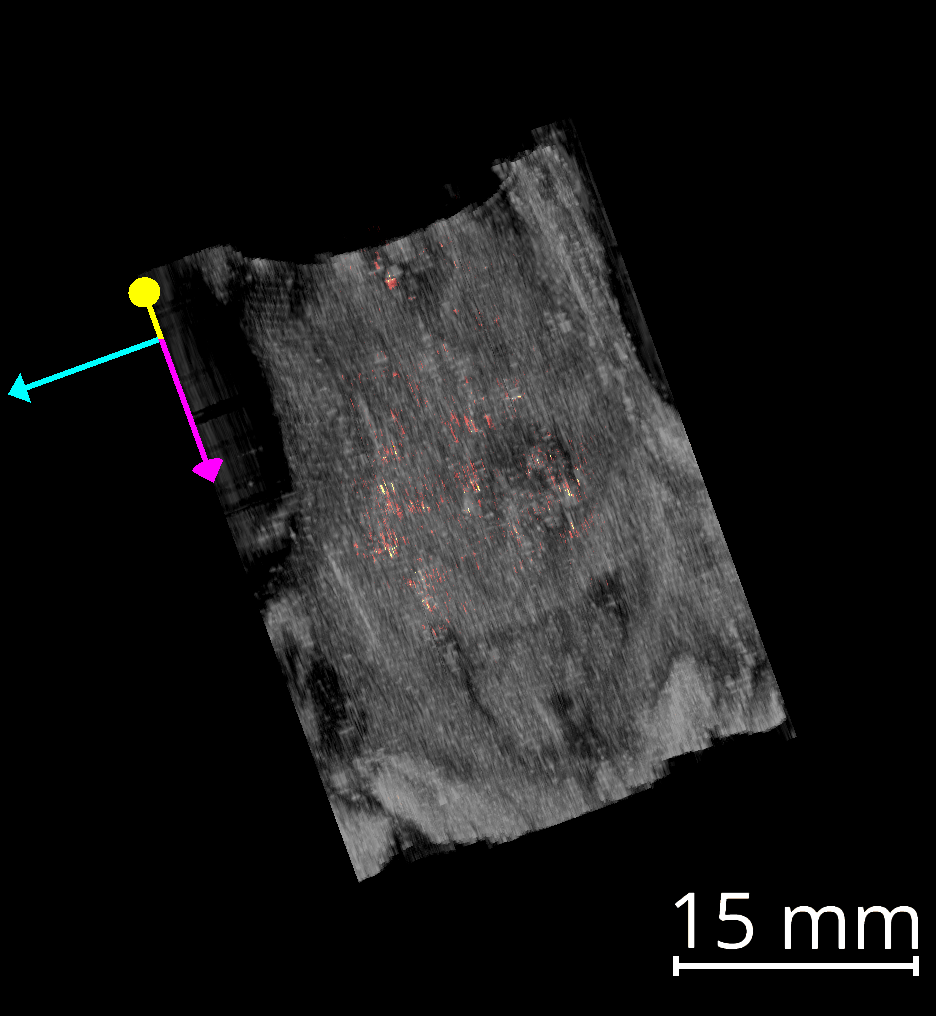

Supplement: Supplementary file 7 — Supplementary Video 5 [file 41467_2025_62569_MOESM7_ESM.gif]
