# Supplementary material for: Probiotic acoustic biosensors for noninvasive imaging of gut inflammation
Source: Nat Commun. 2025 Aug 25;16:7931. doi: 10.1038/s41467-025-62569-1 (PMC12379287; doi:10.1038/s41467-025-62569-1)
Supplement: Supplementary file 9 — Reporting Summary [file 41467_2025_62569_MOESM9_ESM.pdf]

nature portfolio

Corresponding author(s): Mikhail G. Shapiro

Last updated by author(s): June 20, 2025

Reporting Summary

Nature Portfolio wishes to improve the reproducibility of the work that we publish. This form provides structure for consistency and transparency in reporting. For further information on Nature Portfolio policies, see our Editorial Policies and the Editorial Policy Checklist.

Statistics

For all statistical analyses, confirm that the following items are present in the figure legend, table legend, main text, or Methods section.

n/a	Confirmed	
☐	☒	The exact sample size (*n*) for each experimental group/condition, given as a discrete number and unit of measurement
☐	☒	A statement on whether measurements were taken from distinct samples or whether the same sample was measured repeatedly
☐	☒	The statistical test(s) used AND whether they are one- or two-sided<br>*Only common tests should be described solely by name; describe more complex techniques in the Methods section.*
☒	☐	A description of all covariates tested
☒	☐	A description of any assumptions or corrections, such as tests of normality and adjustment for multiple comparisons
☐	☒	A full description of the statistical parameters including central tendency (e.g. means) or other basic estimates (e.g. regression coefficient) AND variation (e.g. standard deviation) or associated estimates of uncertainty (e.g. confidence intervals)
☐	☒	For null hypothesis testing, the test statistic (e.g. *F*, *t*, *r*) with confidence intervals, effect sizes, degrees of freedom and *P* value noted<br>*Give P values as exact values whenever suitable.*
☒	☐	For Bayesian analysis, information on the choice of priors and Markov chain Monte Carlo settings
☒	☐	For hierarchical and complex designs, identification of the appropriate level for tests and full reporting of outcomes
☒	☐	Estimates of effect sizes (e.g. Cohen's *d*, Pearson's *r*), indicating how they were calculated

Our web collection on statistics for biologists contains articles on many of the points above.

Software and code

Policy information about availability of computer code

Data collection	We used MATLAB (version 2017b, Mathworks) custom scripts, with functions provided by the Vantage 4.0.0 system (Verasonics), to acquire ultrasound images. Optical density of samples was measured using the NanoDrop 2000c software (version 1.5, Thermo Fisher Scientific). Fluorescence and opacity images were taken using a Bio-Rad ChemiDoc MP gel imager with Image Lab (version 6.1.0, Bio-Rad Laboratories). Flow cytometry data was acquired using a MACSQuant VYB with MACSQuantify software (version 2.11.1907.19925, Miltenyi Biotec). Ion chromatography mass spectrometry (ICMS) data was acquired using a Dionex Integrion HPIC system with Chromeleon software (version 7.2.10, ThermoFisher Scientific). Ultrasound data acquisition and analysis code are available on the Shapiro Lab GitHub at https://github.com/shapiro-lab in the "Probiotic-GI-Imaging" repository (https://doi.org/10.5281/zenodo.15703247).
Data analysis	We used MATLAB (2021a, Mathworks), Python (version 3.11.5; packages Napari 0.4.19, Numpy 1.26.4, SciPy 1.12.0, and Scikit-image 0.22.0), ImageJ (version 1.54f, NIH), and Prism (version 6, Graphpad) for data and image analysis and plotting. Cytoflow (version 1.2) with custom Python (version 3.8.12; packages Matplotlib 3.5.0, HoloViews 1.17.1, Bokeh 2.4.3, Numpy 1.21.2, Pandas 1.3.5, and SciPy 1.7.3) scripts were used to process flow cytometry data. Illustrations were made in Affinity Designer (version 1.10.6.1665, Serif Europe).

For manuscripts utilizing custom algorithms or software that are central to the research but not yet described in published literature, software must be made available to editors and reviewers. We strongly encourage code deposition in a community repository (e.g. GitHub). See the Nature Portfolio guidelines for submitting code & software for further information.

Data

Policy information about availability of data

All manuscripts must include a data availability statement. This statement should provide the following information, where applicable:

- Accession codes, unique identifiers, or web links for publicly available datasets
- A description of any restrictions on data availability
- For clinical datasets or third party data, please ensure that the statement adheres to our policy

The plasmid constructs generated and used in this study will be made available on Addgene at the time of publication. The bacterial strains, all other materials, and raw unprocessed data are available from the corresponding author upon reasonable request.

Research involving human participants, their data, or biological material

Policy information about studies with human participants or human data. See also policy information about sex, gender (identity/presentation), and sexual orientation and race, ethnicity and racism.

Reporting on sex and gender	This study did not involve human participants, their data, or human-derived biological material.
Reporting on race, ethnicity, or other socially relevant groupings	*Please specify the socially constructed or socially relevant categorization variable(s) used in your manuscript and explain why they were used. Please note that such variables should not be used as proxies for other socially constructed/relevant variables (for example, race or ethnicity should not be used as a proxy for socioeconomic status).*<br>*Provide clear definitions of the relevant terms used, how they were provided (by the participants/respondents, the researchers, or third parties), and the method(s) used to classify people into the different categories (e.g. self-report, census or administrative data, social media data, etc.)*<br>*Please provide details about how you controlled for confounding variables in your analyses.*
Population characteristics	*Describe the covariate-relevant population characteristics of the human research participants (e.g. age, genotypic information, past and current diagnosis and treatment categories). If you filled out the behavioural & social sciences study design questions and have nothing to add here, write "See above."*
Recruitment	*Describe how participants were recruited. Outline any potential self-selection bias or other biases that may be present and how these are likely to impact results.*
Ethics oversight	*Identify the organization(s) that approved the study protocol.*

Note that full information on the approval of the study protocol must also be provided in the manuscript.

Field-specific reporting

Please select the one below that is the best fit for your research. If you are not sure, read the appropriate sections before making your selection.

☒ Life sciences ☐ Behavioural & social sciences ☐ Ecological, evolutionary & environmental sciences

For a reference copy of the document with all sections, see nature.com/documents/nr-reporting-summary-flat.pdf

Life sciences study design

All studies must disclose on these points even when the disclosure is negative.

Sample size	The numbers of biological and technical replicates were chosen based on preliminary experiments, so as to provide sufficient power for statistical comparison.
Data exclusions	No replicates were excluded, but BURST ultrasound acquisitions which captured breathing movements for portions of in vivo GI scans were excluded from downstream analyses; no other data were excluded.
Replication	Replicates are reported in the figure legends.
Randomization	Animals were randomly distributed into cages and ear-punched by animal care staff. Cages of animals were randomly chosen for different conditions (e.g. inflammation induction versus healthy controls). For non-animal experiments, randomization was not necessary because the different strains being compared received the same treatment.
Blinding	For histological analyses, an external pathologist performed blinded grading of tissue samples. For other analyses, blinding was not necessary because data collection, processing, and analysis methods were quantitative and identical across experimental groups.

Reporting for specific materials, systems and methods

We require information from authors about some types of materials, experimental systems and methods used in many studies. Here, indicate whether each material, system or method listed is relevant to your study. If you are not sure if a list item applies to your research, read the appropriate section before selecting a response.

Materials & experimental systems

n/a	Involved in the study
x	Antibodies
x	Eukaryotic cell lines
x	Palaeontology and archaeology
	x Animals and other organisms
x	Clinical data
x	Dual use research of concern
x	Plants

Methods

n/a	Involved in the study
x	ChIP-seq
	x Flow cytometry
x	MRI-based neuroimaging

Animals and other research organisms

Policy information about studies involving animals; ARRIVE guidelines recommended for reporting animal research, and Sex and Gender in Research

Laboratory animals	All mice were 6-10 week-old female Balb/c or male C57BL/6 mice obtained from Jackson Labs. Animal housing room temperatures were monitored at all times and maintained between 71 and 75 degrees F. Humidity was maintained between 30-70%. Light intensity and light cycle timing were carefully regulated and monitored in Caltech laboratory animal facilities. Automated light timers ensured a consistent light-dark cycle with 13 hours on and 11 hours off.
Wild animals	This study did not involve wild animals.
Reporting on sex	Female Balb/c mice were used for initial in vivo experiments involving induction of gas vesicle expression in the GI tract with externally-supplied compounds (Fig. 4, S9, S10, S11, S21) due to their ease of handling and housing. Male C57BL/6 mice were used for all in vivo experiments involving inflammation (Fig. 5, S13, S14, S16, S17, S18, S19, S20) because, although they are more difficult to handle and house, male C57BL/6 mice have been reported to be more susceptible to certain types of chemically-induced inflammation than female mice or than mice of other strains (Koelink PJ and te Velde AA. Mistakes in mouse models of IBD and how to avoid them. UEG Education 2016: 16: 11–14. https://ueg.eu/a/182). So male C57BL/6 mice were exclusively used for experiments involving inflammation to provide a consistent response for this animal model of inflammation, although future studies should incorporate female mice and other strains. The numbers of these mice are indicated in the figure legends.
Field-collected samples	This study did not involve samples collected from the field.
Ethics oversight	Institutional Animal Care and Use Committee (IACUC) of the California Institute of Technology (Caltech) for animal experiments.

Note that full information on the approval of the study protocol must also be provided in the manuscript.

Plants

Seed stocks	This study did not involve plants.
Novel plant genotypes	*Describe the methods by which all novel plant genotypes were produced. This includes those generated by transgenic approaches, gene editing, chemical/radiation-based mutagenesis and hybridization. For transgenic lines, describe the transformation method, the number of independent lines analyzed and the generation upon which experiments were performed. For gene-edited lines, describe the editor used, the endogenous sequence targeted for editing, the targeting guide RNA sequence (if applicable) and how the editor was applied.*
Authentication	*Describe any authentication procedures for each seed stock used or novel genotype generated. Describe any experiments used to assess the effect of a mutation and, where applicable, how potential secondary effects (e.g. second site T-DNA insertions, mosiacism, off-target gene editing) were examined.*

Flow Cytometry

Plots

Confirm that:

- [x] The axis labels state the marker and fluorochrome used (e.g. CD4-FITC).
- [x] The axis scales are clearly visible. Include numbers along axes only for bottom left plot of group (a 'group' is an analysis of identical markers).
- [x] All plots are contour plots with outliers or pseudocolor plots.
- [x] A numerical value for number of cells or percentage (with statistics) is provided.

Methodology

Sample preparation	For flow cytometry of bacterial cells from in vitro samples (liquid cultures and patches from plates), cells were diluted into PBS + 0.5% (w/v) BSA + 1 mg/mL chloramphenicol on ice to target 10^6 cells/mL. For flow cytometry of fecal samples, feces were homogenized in PBS on ice, diluted to 50 mg/mL feces in ice-cold PBS + 1 mg/mL chloramphenicol, filtered through a 40 um membrane, and incubated at 37C and 250 rpm for one hour to allow fluorophore maturation while protein synthesis was inhibited by the chloramphenicol. The fecal samples were further diluted to 2.5 mg/mL feces in ice-cold PBS + 0.5% (w/v) BSA + 1 mg/mL chloramphenicol before being run on the cytometer.
Instrument	MACSQuant VYB (Miltyeni Biotec)
Software	MACSQuantify software (version 2.11.1907.19925, Miltenyi Biotec) was used for flow cytometry data collection. Cytoflow (version 1.2) with custom Python (version 3.8.12; packages Matplotlib, HoloViews, Bokeh, Numpy, Pandas, and SciPy) scripts were used to analyze flow cytometry data.
Cell population abundance	Flow cytometry was only used for analysis, not for sorting.
Gating strategy	Events were gated on FSC-A and SSC-A characteristic of E. coli and on positive mCherry fluorescence (see Fig. S22).

☒ Tick this box to confirm that a figure exemplifying the gating strategy is provided in the Supplementary Information.
