# Supplementary material for: Probiotic acoustic biosensors for noninvasive imaging of gut inflammation
Source: Nat Commun. 2025 Aug 25;16:7931. doi: 10.1038/s41467-025-62569-1 (PMC12379287; doi:10.1038/s41467-025-62569-1)
Supplement: Supplementary file 10 — Transparent Peer Review file [file 41467_2025_62569_MOESM10_ESM.pdf]

nature portfolio

Peer Review File

Probiotic acoustic biosensors for noninvasive imaging of gut inflammation

Corresponding Author: Professor Mikhail Shapiro

This manuscript has been previously reviewed at another journal. This document only contains information relating to versions considered at Nature Communications.

Version 0:

Reviewer comments:

Reviewer #2

(Remarks to the Author)
The authors adequately addressed the reviewer concerns and made attempts to clarify the limitations of the current proof-of-concept demonstration and provide suggestions for future work. There are no outstanding issues that the reviewer has about the manuscript.

Reviewer #3

(Remarks to the Author)
The authors have addressed all my concerns about the previous submission.

Open Access This Peer Review File is licensed under a Creative Commons Attribution 4.0 International License, which permits use, sharing, adaptation, distribution and reproduction in any medium or format, as long as you give appropriate credit to the original author(s) and the source, provide a link to the Creative Commons license, and indicate if changes were made.

In cases where reviewers are anonymous, credit should be given to 'Anonymous Referee' and the source.
The images or other third party material in this Peer Review File are included in the article's Creative Commons license, unless indicated otherwise in a credit line to the material. If material is not included in the article's Creative Commons license and your intended use is not permitted by statutory regulation or exceeds the permitted use, you will need to obtain permission directly from the copyright holder.
To view a copy of this license, visit https://creativecommons.org/licenses/by/4.0/
